# Metabolic and evolutionary responses of *Clostridium thermocellum* to genetic interventions aimed at improving ethanol production

**DOI:** 10.1186/s13068-020-01680-5

**Published:** 2020-03-10

**Authors:** Evert K. Holwerda, Daniel G. Olson, Natalie M. Ruppertsberger, David M. Stevenson, Sean J. L. Murphy, Marybeth I. Maloney, Anthony A. Lanahan, Daniel Amador-Noguez, Lee R. Lynd

**Affiliations:** 1 Thayer School of Engineering, Dartmouth College, Hanover, NH 03755 USA; 2 Department of Bacteriology, University of Wisconsin, Madison, WI 53706 USA; 3 The Center for Bioenergy Innovation, Oak Ridge National Laboratory, Oak Ridge, TN 37831 USA

**Keywords:** *Clostridium thermocellum*, Adaptive laboratory evolution, Ethanol, Isobutanol, Cellulose utilization, Chemostat cultivation, Amino acid secretion, *adhE*, *hfsB*

## Abstract

**Background:**

Engineering efforts targeted at increasing ethanol by modifying the central fermentative metabolism of *Clostridium thermocellum* have been variably successful. Here, we aim to understand this variation by a multifaceted approach including genomic and transcriptomic analysis combined with chemostat cultivation and high solids cellulose fermentation. Three strain lineages comprising 16 strains total were examined. Two strain lineages in which genes involved in pathways leading to organic acids and/or sporulation had been knocked out resulted in four end-strains after adaptive laboratory evolution (ALE). A third strain lineage recapitulated mutations involving *adhE* that occurred spontaneously in some of the engineered strains.

**Results:**

Contrary to lactate dehydrogenase, deleting phosphotransacetylase (*pta*, acetate) negatively affected steady-state biomass concentration and caused increased extracellular levels of free amino acids and pyruvate, while no increase in ethanol was detected. Adaptive laboratory evolution (ALE) improved growth and shifted elevated levels of amino acids and pyruvate towards ethanol, but not for all strain lineages. Three out of four end-strains produced ethanol at higher yield, and one did not. The occurrence of a mutation in the *adhE* gene, expanding its nicotinamide-cofactor compatibility, enabled two end-strains to produce more ethanol. A disruption in the *hfsB* hydrogenase is likely the reason why a third end-strain was able to make more ethanol. RNAseq analysis showed that the distribution of fermentation products was generally not regulated at the transcript level. At 120 g/L cellulose loadings, deletions of *spo0A*, *ldh* and *pta* and adaptive evolution did not negatively influence cellulose solubilization and utilization capabilities. Strains with a disruption in *hfsB* or a mutation in *adhE* produced more ethanol, isobutanol and 2,3-butanediol under these conditions and the highest isobutanol and ethanol titers reached were 5.1 and 29.9 g/L, respectively.

**Conclusions:**

Modifications in the organic acid fermentative pathways in *Clostridium thermocellum* caused an increase in extracellular pyruvate and free amino acids. Adaptive laboratory evolution led to improved growth, and an increase in ethanol yield and production due a mutation in *adhE* or a disruption in *hfsB*. Strains with deletions in *ldh* and *pta* pathways and subjected to ALE demonstrated undiminished cellulolytic capabilities when cultured on high cellulose loadings.

## Background

Metabolic engineering may be idealized as a 3-step process involving quantitative analysis of metabolism, genetic interventions aimed at achieving a desired result, and experimentally validating that the result has been achieved. In practice, genetic interventions often lead to responses other than those targeted and/or induce new selective pressures, which cause the host strain to evolve. Such induced evolution sometimes enhances performance in desired ways—e.g., when a kinetic bottleneck is spontaneously relieved as a result of a mutation—and sometimes it diminishes performance. Understanding metabolic and evolutionary responses to genetic interventions is an instructive and often necessary part of the design–build–test cycle of metabolic engineering [1]. Engineering thermophilic bacteria to produce ethanol provides illustrative examples of such responses, and is considered here.

The anaerobic thermophilic bacterium *Clostridium thermocellum* is a promising candidate for the conversion of lignocellulosic feedstocks to biofuels due to its native ability to solubilize cellulose [2–4]. A major impediment to commercialization is the low ethanol yield of the wild-type organism, typically 12–34% of the maximum theoretical [5]. Aiming to replicate the success realized with the non-cellulolytic hemicellulose-fermenting thermophile *Thermoanaerobacterium saccharolyticum* [6, 7], initial metabolic engineering efforts aimed at increasing ethanol yield in *C. thermocellum* focused on deleting pathways for carbon flux to lactate and acetate. Prominent examples of this approach are described in the papers of Argyros et al. [8] and van der Veen et al. [9]. In both studies, started with the same parent stain (LL345), pathway disruption did not immediately result in changes in ethanol yield, and adaptive laboratory evolution (ALE) was used to give the microorganisms a chance to adjust their metabolism in response to genetic interventions. Whereas ALE resulted in substantially increased ethanol yield in the case of a strain engineered by Argyros et al., it resulted in higher yield of amino acids (and not ethanol) in the end-strain described in van der Veen et al.

In this paper, we aimed to identify the mechanisms underlying the divergent ethanol production phenotypes of the lineages described in Argyros et al. and in van der Veen et al. to evaluate the role and impact of ALE on engineered strains, and to evaluate the extent to which engineering to increase product yield impacts cellulose fermentation capability. We applied an approach that was intended to provide us with multiple types of information; whole-genome sequencing, RNAseq and it included three different cultivation approaches each with different cultivation related fermentation data.

## Results and discussion

### Strain lineages and analysis

**Fig. 1:**
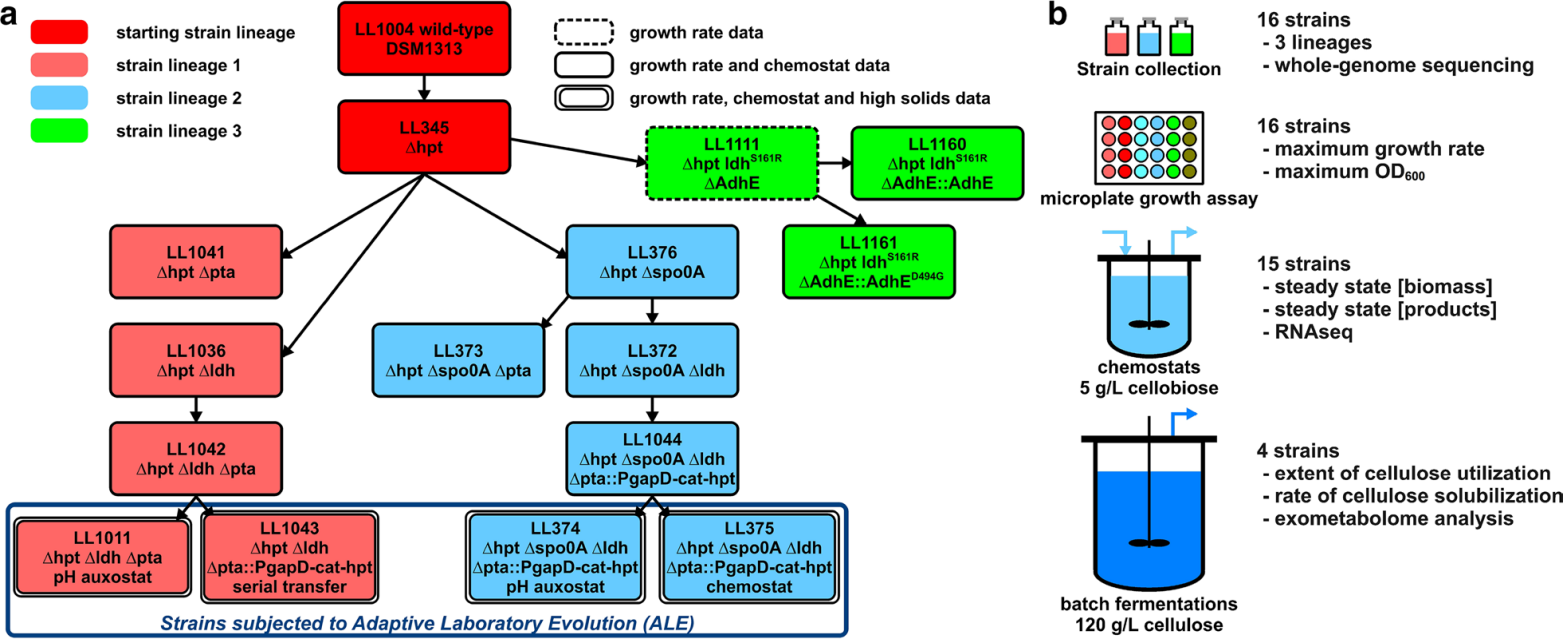
Overview of the strain lineages and data associated with each strain (**a**) and overview of the research approach and data analysis in this paper (**b**). A total of 16 strains were analyzed, covering three different lineages. For each stage of the analysis different data sets were collected. Four strains (LL1011, LL1043, LL374 and LL375) have been subjected to Adaptive Laboratory Evolution, as indicated in the figure. For description of the strain, the adaptation, data generation and analysis see “Methods” section

Starting with *C. thermocellum* strain DSM1313, the three strain lineages discussed here were developed either at Mascoma Corporation or at the Lynd laboratory by a combination of targeted genetic interventions and adaptive laboratory evolution (ALE) as described in “Methods” section (the strain developing process as such is not part of the effort described here and can be found elsewhere). The lineages of the strains studied are presented in Fig. 1. Strain LL345 was developed from wild-type *C. thermocellum* (DSM 1313) by deleting the *hpt* gene, thereby allowing counter-selection using 8AZH (8-azahypoxanthine) useful for making unmarked genetic modifications. No change in the distribution of fermentation products was observed upon deletion of the *hpt* gene. In lineage 1, the genes for lactate and acetate production were deleted and the strains were evolved for faster growth using a pH auxostat (strain LL1011) or serial transfer (strain LL1043). In lineage 2, the *spo0A* gene was deleted to disrupt sporulation [10], then the genes for lactate and acetate production were deleted, and strains were evolved for faster growth using a pH auxostat (strain LL374) or chemostat (strain LL375). Lineage 3 involved mutations in the *adhE* gene; wild-type *adhE* was deleted from LL345 resulting in strain LL1111. The wild-type gene was reintroduced in strain LL1160, and a D494G mutant *adhE* was reintroduced in strain LL1161. Lineage 3 also contains a mutation in the *ldh* gene (ldh^S161R^) that appeared during the creation of strain LL1111 from LL345 as discussed in Lo et al. [11].

All strains were whole-genome sequenced and tested for growth by microplate assay (see Fig. 1). All but strain LL1111 were grown in chemostats on 5 g/L cellobiose where data for fermentation products and biomass concentration were collected and sampled for RNAseq. Finally, four strains that underwent ALE were cultivated on 120 g/L cellulose where the exometabolome was sampled over time and data for cellulose solubilization were collected.

### Growth rate measurements

**Fig. 2:**
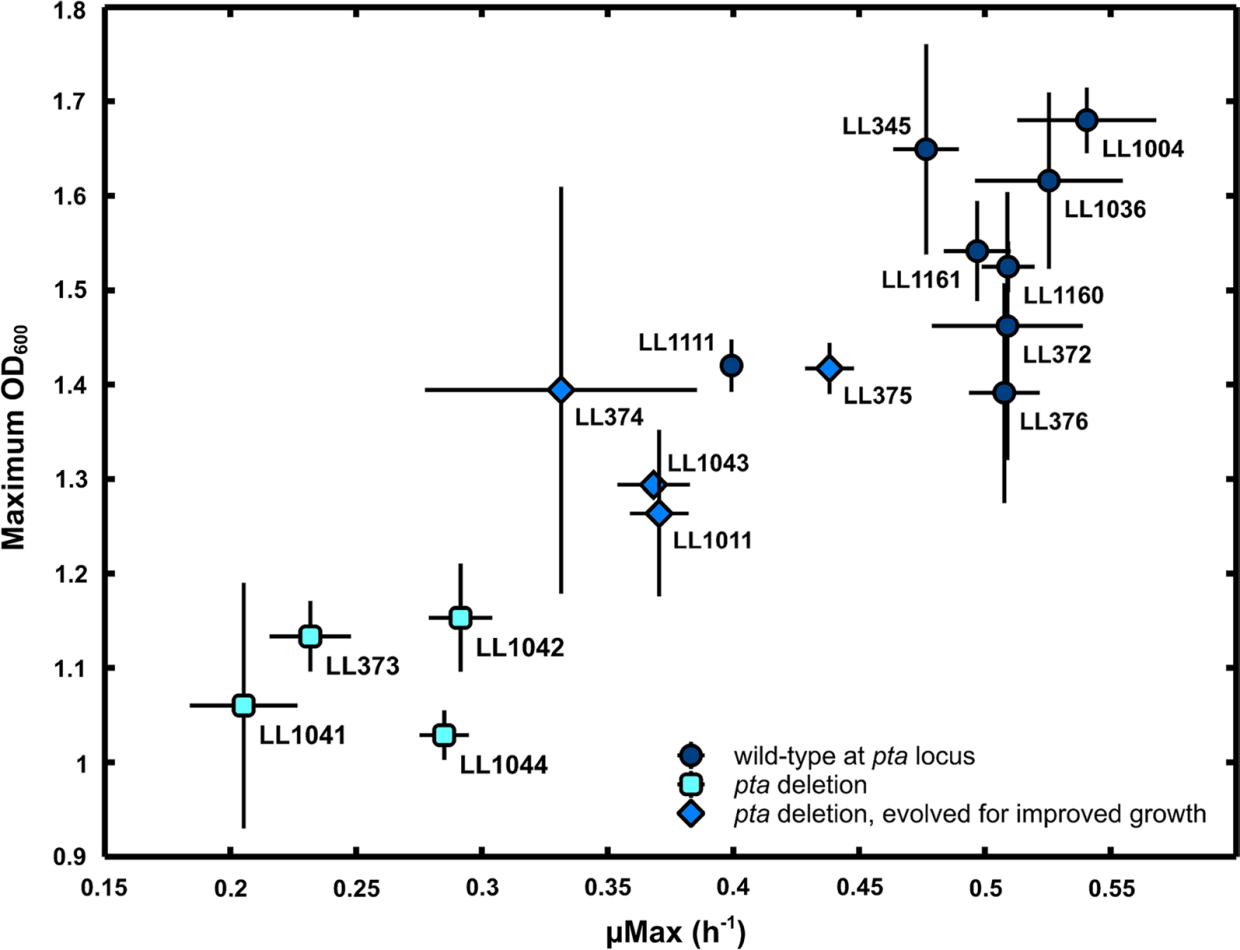
The maximum growth rate versus the maximum cell density as measured by OD_600_ for strains described in this study. Strains were cultured in a microplate placed in a plate reader, incubated at 55 °C on modified low-carbon medium with 5 g/L cellobiose. Error bars represent one standard deviation, *n* ≥ 3 cultures

Prior to characterizing growth in chemostats or under high cellulose loadings, all strains were cultured in microplates on a plate reader to determine maximum growth rate (*µ*_max_) and biomass formation as measured by OD_600_ (Fig. 2). In general, strains exhibiting a higher maximum OD_600_ also exhibited a higher *µ*_max_. The factor that showed the biggest influence on both variables was the deletion of the *pta* gene. Deleting the *pta* gene had a significant negative effect on both the growth rate as well as the maximum OD as measured under batch conditions in the microplate. However, within the group of *pta* deletion strains, the evolved strains (LL374, LL375, LL1011 and LL1043) exhibited an improvement in growth rate after adaptation. To understand the effect of *pta* deletions on fermentation behavior, without the confounding effect of growth rate, we performed steady-state chemostat fermentations.

### Chemostat cultivation and changes in metabolic flux

**Fig. 3:**
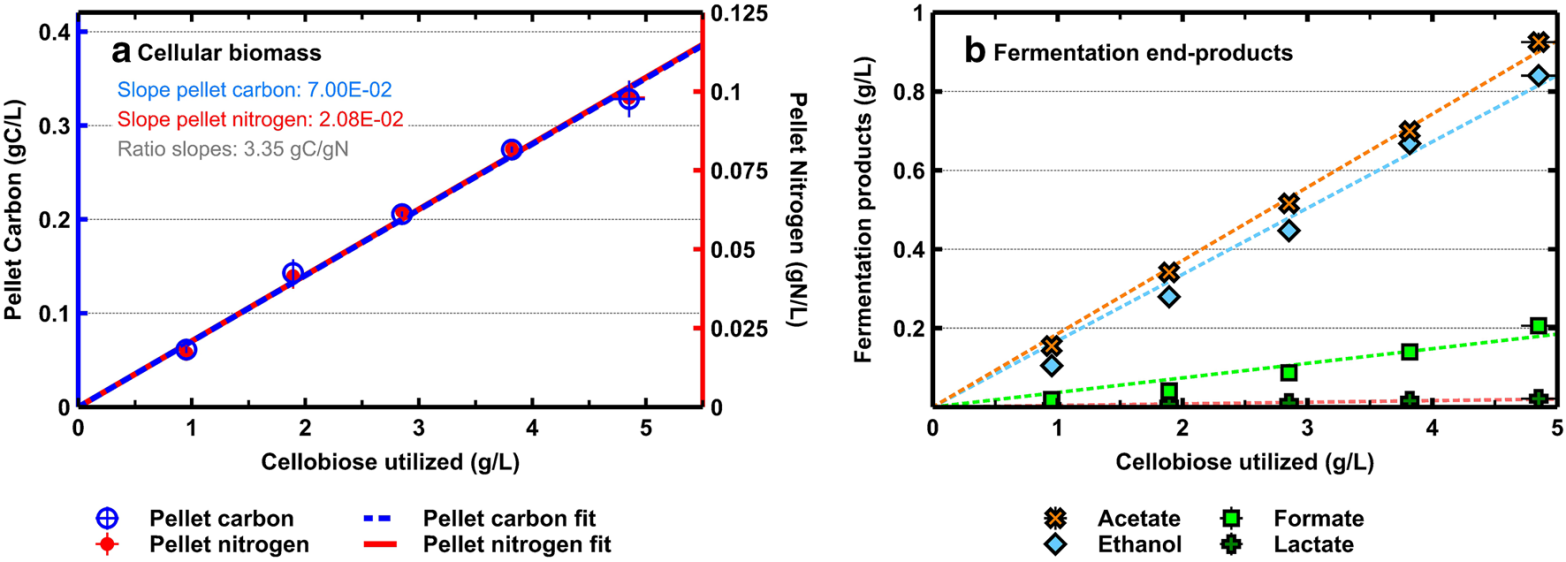
Cellular biomass (**a**) and fermentation end-product (**b**) formation for five different cellobiose loadings at dilution rate of 0.1 h^−1^ for *Clostridium thermocellum* wild-type (LL1004) at steady state. Both cell yield and product formation have a linear relation with the amount of substrate utilized. Steady-state cell concentration is represented by both pellet carbon and pellet nitrogen, which both have a linear relation to cellobiose utilized. Error bars represent one standard deviation

To ensure the medium was able to support growth in chemostats at a *D* = 0.1 h^−1^, and that the culture was carbon limited, wild-type *C. thermocellum* (LL1004) was cultured at 5 different cellobiose concentrations and sampled at steady state. Figure 3 shows a linear relation between cell concentration and carbohydrate loading. Cell concentration is expressed in terms of pellet carbon and nitrogen, which are linearly related to cellobiose utilized and to each other at a ratio of 3.35 g carbon/g nitrogen. The fermentation end-products ethanol, acetate, formate and lactate also are linearly correlated with substrate utilized, which indicates there is no limitation other than carbon from cellobiose.

To understand the effect of the targeted mutations and subsequent adaptation on fermentation behavior, 15 strains were grown in chemostats under carbon-limiting conditions on 5 g/L cellobiose at a residence time (RT) of 10 h (dilution rate of 0.1 h^−1^). Steady state was verified by on-line in situ OD measurements, and the chemostats were sampled after ≥ 4 residence times and again after ≥ 7 residence times for at least duplicate, independently inoculated chemostats. Samples were analyzed for cellular biomass and major fermentation products (lactate, acetate, formate and ethanol) as well as pyruvate and free amino acids. The hydrogen concentration in the headspace was also determined and samples were checked for presence of intermediate metabolites like fumarate and malate as well as isobutanol and pyroglutamate. Non-normalized data can be found in Additional file 1: Table S1.

**Fig. 4:**
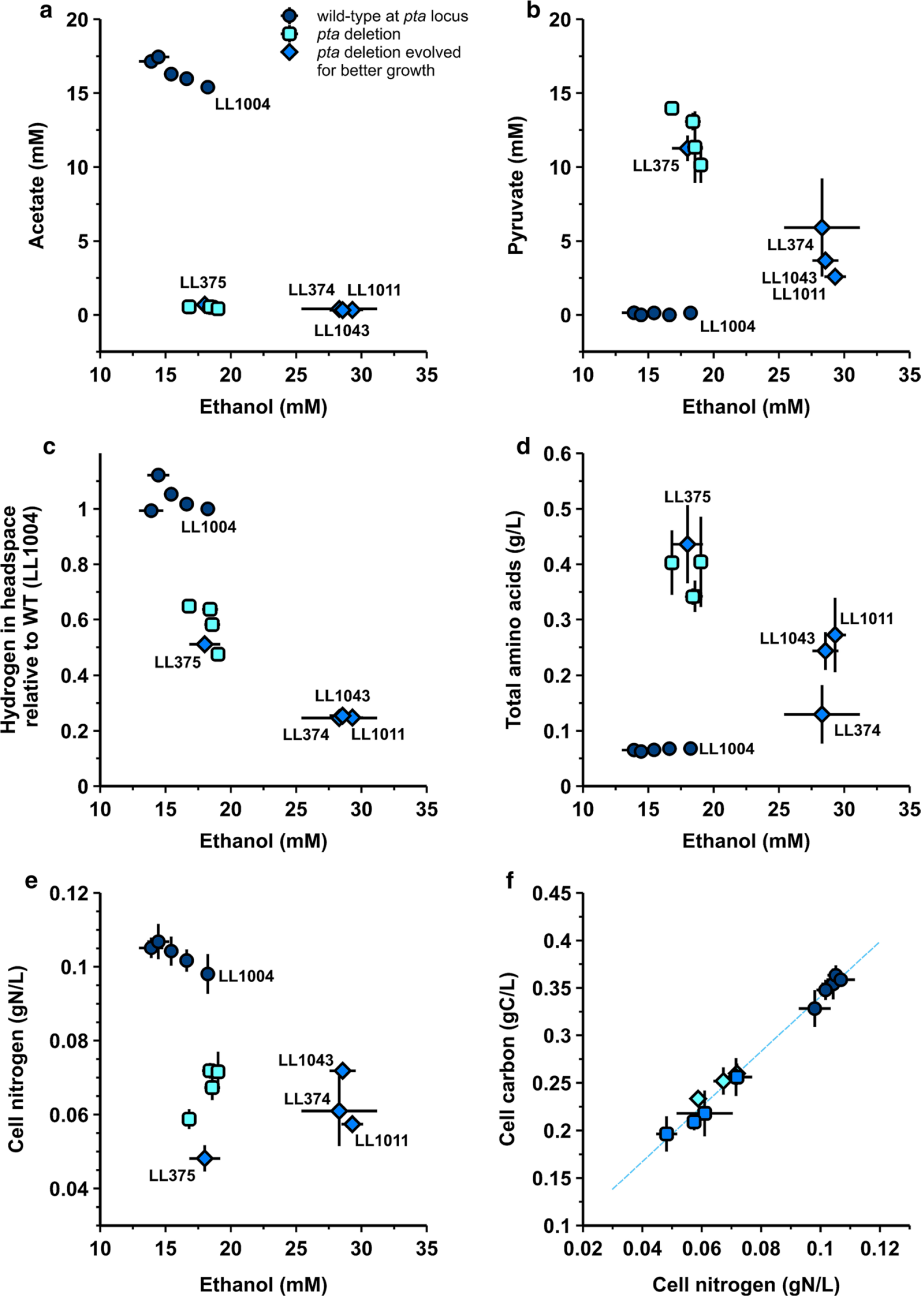
A comparison of steady-state production level of ethanol vs. acetate (**a**), pyruvate (**b**), hydrogen (**c**), total amino acids (**d**) and cell nitrogen (**e**, a proxy for cell biomass) for strain lineages 1 and 2. **f** Shows data for cell carbon to cell nitrogen for the same data set for every strain. Strains with wild-type *pta* are represented by round shaped dark blue data points, strains with *pta* deleted (unmarked deletion or merodiploid) are square shaped light blue data points and strains with *pta* deleted but evolved for improved growth are diamond shaped blue data points. The evolved strains with *pta* deleted (LL374, LL375, LL1011 and LL1043) are indicated with their strain name as is wild-type (LL1004). Error bars represent one standard deviation, *n* ≥ 4. This figure does not include data for strains LL1111, LL1160 and LL1161, and the data used here is not normalized for differences in cellobiose feed concentration or for measured dilution rate

Measured dilution rates ranged from 0.092 h^−1^ (LL375) to 0.109 h^−1^ (LL1036). The measured substrate concentration in the feed ranged from 4.69 to 4.88 g/L (see also Additional file 1: Table S1). Figure 4 shows 6 panels representing steady-state production levels of acetate (panel a), pyruvate (panel b), hydrogen (panel c), amino acids (panel d), cell nitrogen (panel e) versus that of ethanol. Panel f represents the ratio of cell carbon to cell nitrogen.

Panels a to e show that deleting either *hpt* and *spo0A* and/or *ldh* has minimal effect on ethanol and cell biomass at steady state; strains containing only these deletions (LL345, LL1036, LL376 and LL372) shown in dark blue circles are grouped with LL1004 (wild-type). Deleting *pta* (light blue squares, strains LL1041, LL1042, LL373 and LL1044) results in decreased acetate production and a lower steady-state biomass concentration. However, this decrease is not accompanied by an increase in ethanol; there is instead an increase in pyruvate (b), and free total amino acids (d), and a decrease in hydrogen (c) and cell nitrogen (cell biomass, e). It seems that under the tested conditions the primary response of metabolism after blocking acetate production is an increase in pyruvate and amino acids (with valine, alanine, glutamine and isoleucine being highest detected). Four strains were evolved for improved growth (represented by blue diamonds) as described earlier, and this results in an increase in ethanol (panels a–e) for strains LL374, LL1011 and LL1043 accompanied by a decrease in pyruvate, hydrogen and amino acids. Strain evolution after pta deletion (panel e) does not recover the decreased cell biomass concentration. For all the strains the ratio between cell carbon and nitrogen remains constant (panel f). Steady-state hydrogen (c) decreases further with increasing ethanol concentration due to strain evolution. Out of the four evolved strains with pta deleted, LL375 is the only strain where ethanol production is not improved. Steady-state pyruvate, hydrogen, amino acids and cell nitrogen do not change after adaptation and the strain remains grouped with the unevolved *pta*-deleted strains (in light blue squares).

#### Control of lactate flux

Lactate production in *C. thermocellum* is controlled by the lactate dehydrogenase gene (*ldh*), which is constitutively expressed at high levels, but is allosterically controlled by fructose 1,6-bisphosphate (FBP) [11]. Under normal conditions, *C. thermocellum* ferments C_6_ sugars to mainly acetate and ethanol. When the pathways for these fermentation end-products are blocked, intracellular metabolites accumulate, including FBP, which activates the Ldh enzyme. During fermentation on cellulose, lactate usually appears only after ethanol and acetate formation is well underway [12–15], consistent with a lag in accumulation of FBP in central metabolism. Strains LL1111, LL1160 and LL1161 have the Ldh^S161R^ mutation which de-represses lactate production (i.e., lactate is produced all of the time, even when FBP levels are low), and explains the increased lactate production in these strains [11]. Lactate production from glucose is balanced for electrons. Under the chosen chemostat cultivation conditions, strains with a wild-type *ldh* locus do not produce much lactate, which masked the effect of the *ldh* deletion.

#### Control of acetate flux

**Fig. 5:**
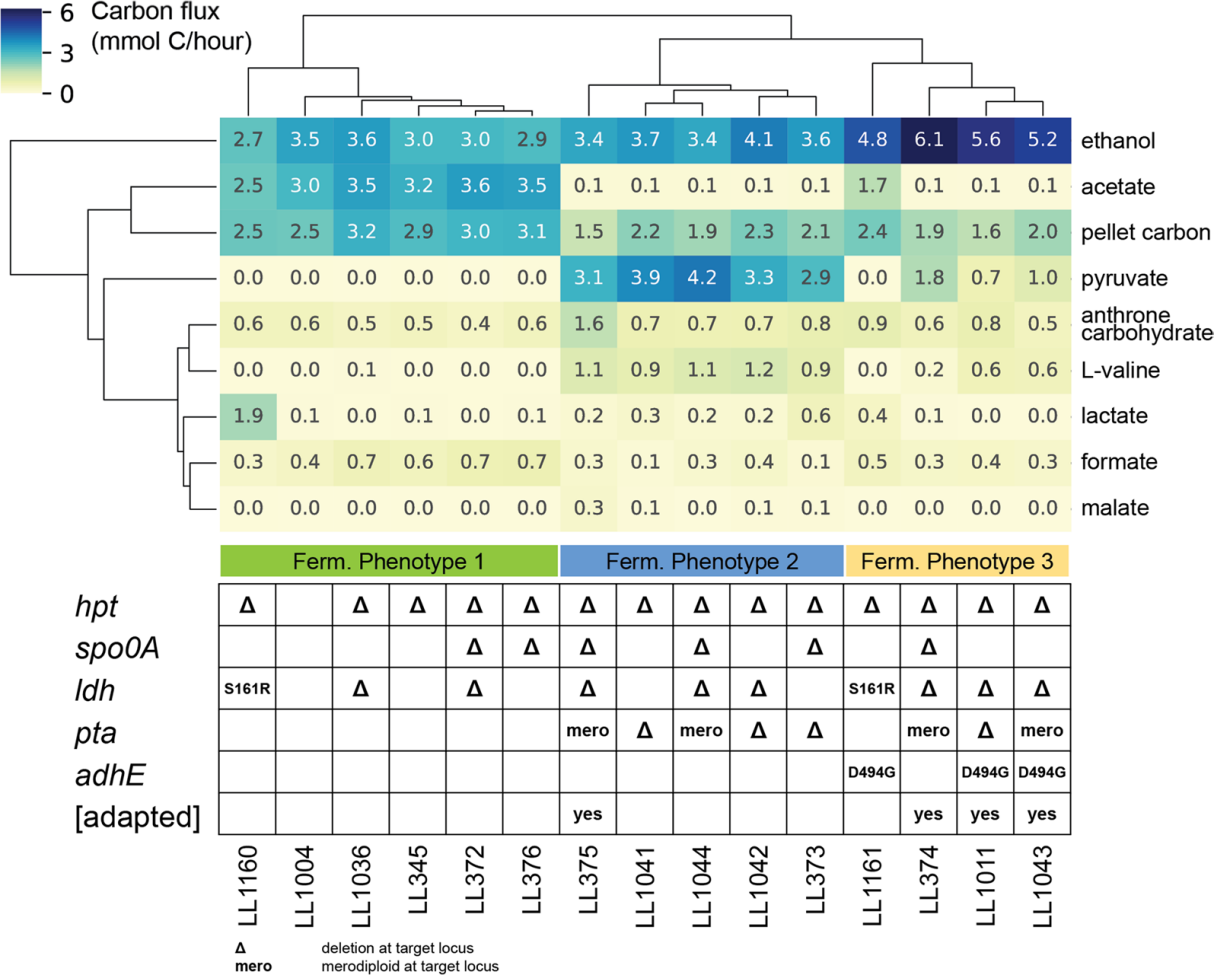
Clustered fermentation data. Units are carbon flux (mmol C-atoms/h). Only products accounting for > 3% of the total carbon flux were included. Hierarchical clustering was performed for both strains and products using an averaged linkage method and Euclidean distance metric. The full set of fermentation data is available in Additional file 1: Table S1

The most significant fermentation phenotype is the change in acetate production upon deletion of the pta gene (Figs. 4 and 5). Strains that are wild type at the pta locus produce about 17 mM acetate. When pta is deleted, acetate production is largely eliminated (traces of acetate may be produced as a side product of other biological reactions). Deleting pta has a negative effect on ATP yield per sugar utilized in anaerobic metabolism. This loss in ATP can affect energetically intense cellular processes like protein and cell synthesis, which in case of *C. thermocellum* and its cellulosome could influence its cellulolytic capabilities. The steady-state cell concentration as shown by pellet carbon or pellet nitrogen values in Figs. 4 and 5 decreases when pta is deleted, and is not recovered after ALE. The decrease in acetate flux corresponds to an increase in pyruvate secretion. During adaptive evolution, pyruvate flux decreases, and ethanol flux increases. Of the four adapted strains (LL374, LL375, LL1011 and LL1043), three of them fit this pattern. The fourth strain (LL375) does not. In this strain adaptive evolution increased the growth rate without affecting the fermentation phenotype.

Compared to lactate, the control of acetate production is more complicated, because the pathway for conversion of glucose to acetate is not balanced for electrons (glucose → 2 acetate + 2 CO_2_ + 2 NADH + 2 Fd_red_). Thus, acetate production has to be accompanied by the production of a reduced product such as ethanol, formate and hydrogen. Under conditions of low hydrogen partial pressure (such as co-culturing *C. thermocellum* with a H_2_-consuming methanogen), the acetate:ethanol ratio can be as high as 8.5:1 [16]. Conversely, increasing the H_2_ partial pressure decreases acetate:ethanol ratio [14, 17–20]. Acetate production can also be decreased by eliminating hydrogen production [21].

#### Hierarchical clustering of carbon fluxes

Performing hierarchical clustering on the carbon fluxes (mmol carbon/h, derived from chemostat fermentation data) for all strains (including lineage 3) and the important metabolites (carbon flux > 3% of total) reveals the same three clusters from Figs. 2 and 4, and gives us additional information about related metabolites. Strains in cluster 1 produce ethanol and acetate in a roughly 1:1 molar ratio. All of the strains in this cluster are wild-type for *pta*. Strains in cluster 2 produce mostly ethanol and pyruvate. Amino acid production is elevated, particularly valine. Acetate production has been eliminated. Pellet carbon is reduced by about 50% compared with cluster 1. All of the strains in this cluster have a *pta* deletion. Strains in cluster 3 produce primarily ethanol (about 50% of the maximum theoretical yield). Pyruvate and amino acid production is lower than cluster 2 (*pta* deletion), but higher than cluster 1 (wild type). This cluster includes 3 of the 4 adapted strains, as well as strain LL1161.

### Sequencing data

Mutations in each strain were identified by whole-genome sequencing (see “Methods” for details). A full list of all mutations is available in Additional file 2: Table S2.

### RNAseq mutations

RNAseq data contain information about mutations, in addition to gene expression, and these mutations were added to the mutations found by whole-genome sequencing. For most strains, the whole-genome sequencing data and RNAseq data showed similar results. For strain LL374, several additional mutations were found using RNAseq that were not observed based on whole-genome resequencing. Re-analysis of the sequencing data showed that many of these missing mutations were present in the sequencing data at a low frequency (~ 20% of reads for a given mutation, which was below the cutoff used for the initial analysis). Since these mutations were present at near 100% frequency in the RNAseq data, this suggests that these mutations belong to a subpopulation that overtook the culture at some point after the whole-genome resequencing.

### Merodiploid pta locus

All of the *pta* deletion strains were created in a manner to allow the removal of selectable markers, resulting in an unmarked deletion. After adaptation, however, three of the four adapted strains (LL374, LL375 and LL1043, but not LL1011) were found to be merodiploid at the *pta* locus (i.e., they have a truncated copy of the *pta* gene, Additional file 3: Figure S3). The merodiploid stage is part of the gene deletion process where two copies of the downstream homology flanking region are present [22]. Inspection of reads that did not map to the *C. thermocellum* chromosome revealed a number of reads that mapped to the P_gapDH_-*cat*-*hpt* cassette from plasmid pMU1817 (accession number MK036504) used to delete the *pta* gene [8]. Re-mapping all of the reads to an in silico reconstruction of the merodiploid locus provides evidence that this configuration is present (sequence data from accession numbers in Table 2). First, there are no reads mapped to the 5′ end of the *pta* gene (this also shows that this gene has been functionally inactivated). Second, the 5′ flanking region and *gapDH* promoter region have twice the read depth of the other regions, suggesting that they are present in two copies each, which is what would be expected for a merodiploid strain (the *gapDH* promoter region is present once at the *gapDH* locus on the chromosome and once in the P_gapDH_-*cat*-*hpt* cassette). Third, there is direct evidence of the P_gapDH_-*cat*-*hpt* cassette integration at the *pta* locus based on paired read data where one read pair maps unambiguously to the cassette and one maps unambiguously to the *pta* locus. Fourth, the size of the *pta* and *gapDH* loci was analyzed by PCR (Additional files 3 and 4: Figures S3 and S4). The PCR data support the conclusions from the Illumina sequencing data, namely that strains LL1043 and LL1044 are merodiploid at the *pta* locus.

**Table 1:** Mutations associated with changes in gene expression

Annotation name	Locus description	Parent	Strain	Fold-change	*p*-value
89 bp upstream of Clo1313_0099	Thiamine pyrophosphate TPP-binding domain-containing protein	LL1036	LL1042	4.33	0.0017
10 bp upstream of Clo1313_0779	Copper amine oxidase-like domain-containing protein	LL1044	LL374	0.47	0.0003
81 bp upstream of Clo1313_1055	Major facilitator superfamily MFS_1	LL1044	LL375	2.29	0.0020
8 bp upstream of Clo1313_1397	Copper amine oxidase-like domain-containing protein	LL1044	LL374	0.47	0.0010
14 bp upstream of Clo1313_1989	VTC domain	LL345	LL376	0.52	0.0001
197 bp upstream of Clo1313_2323	ABC transporter related	LL1044	LL374	2.52	0.0005

In the case of strain lineage 1 the *pta* locus shows an unmarked deletion in strain LL1042. Strain LL1011, which is derived from LL1042, also shows an unmarked deletion. Strain LL1043, however, shows a reversion to the merodiploid genotype. Presumably the merodiploid genotype existed as a small population in strain LL1042 (the sequencing data for strain LL1042 do not show any evidence of this, which suggests this population was < 1% of the total, and this was confirmed by PCR). Furthermore, there must have been a selective advantage for this population to outcompete the unmarked deletion population during the selection for faster growth in the pH auxostat. The merodiploid strain has two changes that might explain this. One change is the presence of the *hpt* gene, which would complement the *hpt* deletion that was introduced to allow 8AZH counter-selection [8]. Another change is increased expression of the acetate kinase (*ack*) gene, presumably via read-through from the P_gapDH_ promoter. The maximum growth rate data only show very minimal advantage for strain LL1043 vs. LL1011 (Fig. 2), so the growth advantage must be specific to conditions used to generate the adapted strains.

In the case of strain lineage 2, the *pta* locus of strain LL1044 is merodiploid, and this genotype was passed on to both LL374 and LL375. This appears to be an intentional choice in strain construction. Van der Veen et al. note that the unmarked ∆*ldh* ∆*pta* strain had a growth defect, and thus the merodiploid strain was chosen for chemostat evolution [9].

#### Convergent evolution mutations

Mutations overrepresented in strains with phenotype 3 (high ethanol) are most likely to actually contribute to that phenotype. There are three genes in this category: Clo1313_0670, *adhE* and Clo1313_2130. Gene Clo1313_0670 is annotated as a zinc/iron permease. In strain LL374, the mutation is 28 bp upstream of the Clo1313_0670 gene, which results in a 63% decrease in expression (*p* = 0.006). In strain LL1011, the mutation is a frameshift. Both of these mutations decrease the activity of this gene. This may be related to ethanol tolerance. A strain of *C. thermocellum* adapted for improved ethanol tolerance acquired a T186M mutation in this gene (ATCC 27405 Cthe_3117 locus) [23].

In the *adhE* gene, strains LL1011 and LL1043 both had the same D494G mutation. Furthermore, this is the only mutation that both strains share, suggesting that this mutation may be responsible for the increase in ethanol production observed in those strains. Furthermore, mutations in *adhE* have been observed in other strains of *C. thermocellum* adapted for increased ethanol tolerance (including the D494G mutation) [23, 24].

Very little is known about Clo1313_2310. In strain LL1043, the gene was mutated by insertion of an IS120 transposon, which likely inactivated the gene.

#### Increased ethanol production in strain LL374

Of the three adapted ∆*ldh* ∆*pta* strains that showed increased ethanol yield (LL374, LL1011 and LL1043), two can be explained by the *adhE*^D494G^ mutation. Since strain LL374 does not have this mutation, we need to consider other explanations. Most strains have fewer than 20 mutations, however strain LL374 has 103 (Additional files 2 and 5: Table S2 and Figure S5). One possible explanation for the increased mutation rate is a mutation in DNA polymerase III (Clo1313_1219). The homolog of Clo1313_1219 in *E. coli* is *dnaE*. Mutations in *dnaE* have been shown to generate mutator phenotypes in *E. coli* [25, 26].

A possible explanation for the increase in ethanol production is the disruption of *hfsB* (hydrogenase-Fe-S, Clo1313_1795). Strain LL374 has two point mutations in this gene, one of which is a frame-shift mutation which would be expected to eliminate activity. Disruption of *hfsB* in *C. thermocellum* has previously been shown to increase ethanol production by 52% (from 37% of theoretical to 55% of theoretical) [27]. This increase is similar to what was observed for strain LL374 and thus is a plausible explanation for the increase in ethanol production.

Other mutations are described and evaluated in Additional file 6: Supplemental text S6.

### Re-introduction of the AdhE^D494G^ mutation

The bifunctional alcohol and aldehyde dehydrogenase AdhE performs the conversion of acetyl-CoA into ethanol by two reductive steps and is essential for ethanol production in *C. thermocellum* [11]. In *C. thermocellum*, AdhE uses NADH as a cofactor for both reductive steps (acetyl-CoA reduction (ALDH) and subsequent acetaldehyde reduction (ADH)). A mutation that causes an aspartate-to-glutamate mutation at position 494 (D494G) has been shown to allow the ADH reaction to use either NADH or NADPH [28]. Mutations in the *adhE* gene have been associated with increased ethanol production in *C. thermocellum* [29] as well as other thermophilic bacteria such as *Thermoanaerobacterium saccharolyticum* [30].

**Fig. 6:**
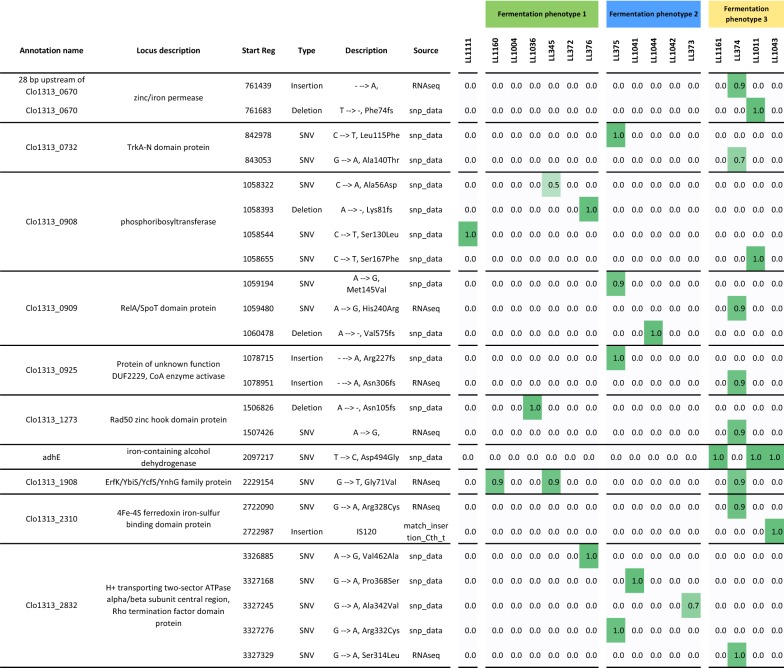
Convergent mutations. Strains are sorted according to the fermentation profiles from Fig. 5 to show genes whose mutations cluster predominantly with a particular phenotype. For example, mutations in the adhE gene seem to be associated with fermentation phenotype 3. Strain LL1111 is not shown in Fig. 5. It produces mainly lactate and is thus given its own fermentation phenotype column. For each mutation, the fraction of reads supporting that mutation is given in the shaded cells. This figure only shows origin mutations, so inherited mutations are not shown. Furthermore, genes are only shown that have more than one origin mutation. A complete list of mutations in each strain is provided in Additional file 2: Table S2. ^a^SNV = single nucleotide variation. ^b^fs = frameshift

In order to identify causal links between a single mutation and phenotype, it is important to reintroduce the mutation in a “clean” strain background, without other secondary mutations that could confound interpretation of the results. Our ability to re-introduce point mutations in *C. thermocellum* is limited by the available genetic tools, and we were thus only able to test a single point mutation. We chose to test the D494G mutation in AdhE, since we know that the *adhE* gene is essential for ethanol production [11], and the mutation was the only mutation shared by both of the adapted strains in lineage 1 (strains LL1011 and LL1043, Fig. 6). To test this hypothesis, we re-introduced this mutation into a strain that was wild type for *ldh* and *pta* (strain LL345). Current genetic tools for *C. thermocellum* do not allow us to directly reintroduce the point mutation. Instead, we had to delete the *adhE* gene (resulting in strain LL1111) and then insert an *adhE* gene with either the wild-type sequence (resulting in strain LL1160) or the D494G mutation (strain LL1161). Since strains LL1160 and LL345 are wild type at the *adhE* locus, comparing their fermentation phenotypes allows us to see the effect of other mutations that occurred during the construction of LL1160. Strain LL1160 had a fermentation profile similar to that of LL345 (i.e., both are found in cluster 1 of the fermentation data, Fig. 5), although lactate production had increased due to a mutation that eliminated fructose 1,6-bisphosphate regulation of the *ldh* gene [11]. Comparing strains LL1160 and LL1161 shows the effect of the D494G mutation, which is to increase ethanol production at the expense of lactate and acetate production (i.e., move from fermentation cluster 1 to cluster 3). This single point mutation can explain about 90% of the increase in ethanol production observed in strains LL1011 and LL1043.

### Gene expression during chemostat growth

Gene expression in each strain was determined by RNAseq (Additional files  7, 8 and 9: Table S7, Table S8 and Table S9). Comparing each evolved strain to a strain that is wild type at the *ldh* and *pta* loci (LL345) showed only a small number of significant differences (Additional file 10: Figure S10).

#### Mutations correlated to changes in gene expression

To identify mutations that were responsible for changes in gene expression, we looked at mutations that occurred upstream of a given gene, and then compared gene expression in that strain with expression in the parent strain. Of the 22 origin mutations that were found to be upstream of a gene, 6 were significant at the *p* = 0.05 level (corrected for 22 multiple tests by the Bonferroni method) (Table 1). Of these mutations, the only one with a distinct fermentation phenotype is the mutation in strain LL1042 upstream of Clo1313_0099, which is the valine biosynthesis operon. It is possible that this mutation explains the increase in valine production in this strain, however there are several other strains with elevated valine production that do not have this mutation.

### Batch cultivation on high cellulose loadings

Under industrially relevant cellulose loadings (≥ 100 g/L cellulose) wild-type *C. thermocellum* demonstrates three significant features. It can solubilize and ferment 80–100 g/L crystalline cellulose [14, 31]. Cell growth stops at around 50% solubilization while the culture continues to solubilize and metabolize the remaining cellulose without apparent effects on rate or extent of solubilization [13]. Lastly, near the point of maximum cell concentration the culture switches to uncoupled metabolism and produces higher alcohols (>C_2_, notably isobutanol and 2,3-butanediol) as well as continues to produce significant amounts of amino acids (notably valine and alanine) [13]. All of these events only become evident when culturing under high cellulose loading conditions, and have been attributed to overflow metabolism around the pyruvate node [13].

**Fig. 7:**
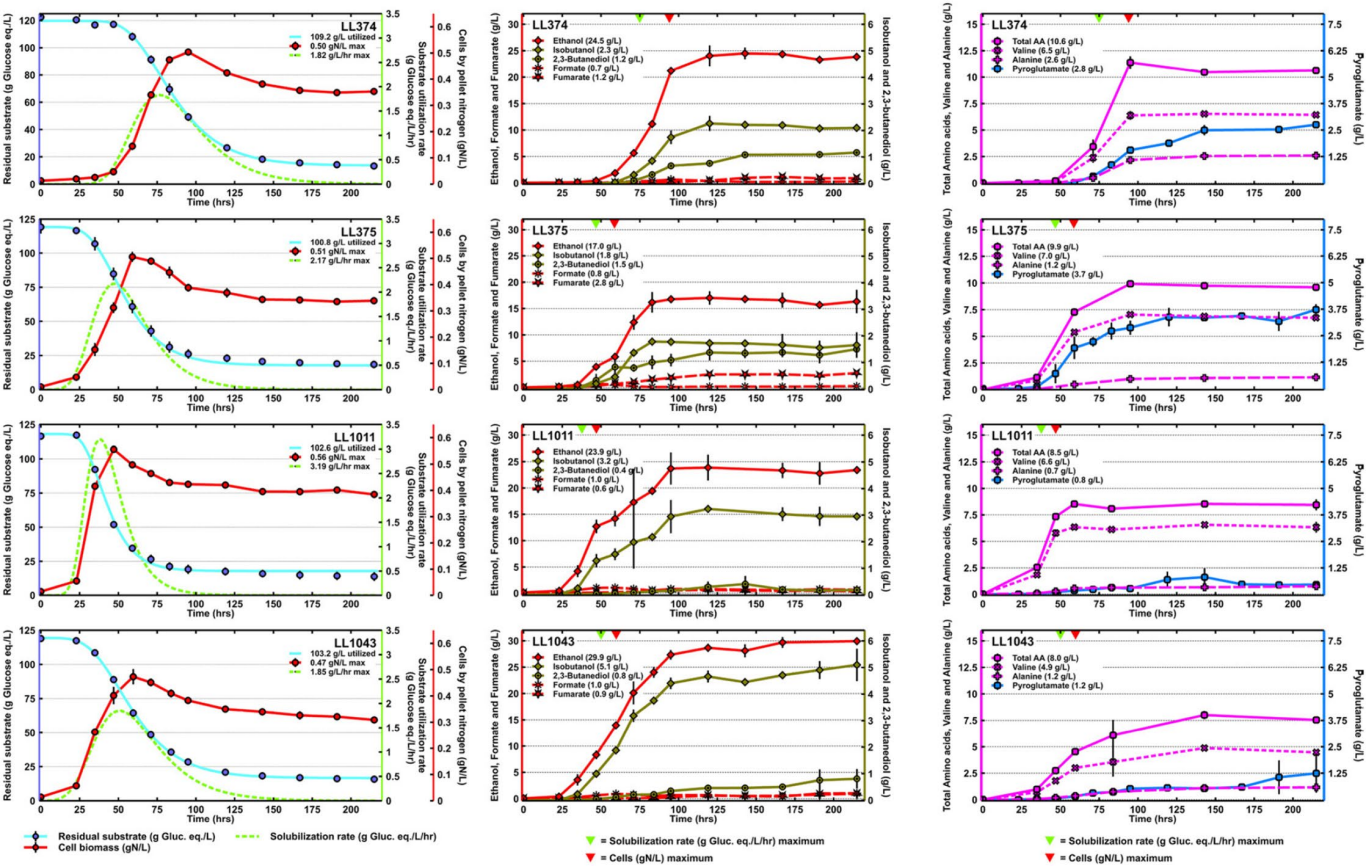
Strains LL374 (∆*hpt* ∆*spo0A* ∆*ldh* ∆*pta*::*PgapD*-*cat*-*hpt* adapted by pH auxostat), LL375 (∆*hpt* ∆*spo0A* ∆*ldh* ∆*pta*::*PgapD*-*cat*-*hpt* adapted by chemostat), LL1011 (∆*hpt* ∆*ldh* ∆*pta* adapted by serial transfer), LL1043 (∆*hpt* ∆*ldh* ∆*pta*::*PgapD*-*cat*-*hpt* adapted by pH auxostat) cultivated on 120 g/L cellulose. Both strains LL1011 and LL1043 have the *adhE*^D494G^ mutation. The left panels show residual cellulose concentration fitted with a sigmoidal curve, the rate of cellulose solubilization and pellet nitrogen concentration as proxy for cells. The middle panel figures show major fermentation products ethanol, isobutanol as well as formate and fumarate. The right panel set shows total amino acids, valine and alanine as well as pyroglutamate. For the middle and right panel sets the arrows on the top y-axis indicate maximum cell concentration (red) and maximum of cellulose solubilization (green). Values in the brackets of the legend of the left panel figures represent the maximum values of the amount cellulose utilized, cell concentration and cellulose solubilization rate (as indicated), values shown in brackets in the legends of the middle and right panels show the maximum average values measured of fermentation metabolites. All data other than the residual cellulose concentration fit curve and the solubilization rate curve are averages from duplicate fermentation runs, and error bars represent ± 1 standard deviation

Wild-type *C. thermocellum* has been shown to be capable of solubilizing between 77.5 and 96.7 g glucose equivalents/liter, at a rate of 2.09–2.88 g gluc eq/L/h when cultivated on up to 103.4 g gluc eq/L [13]. Figure 7, in the left panel of figures, shows the extent of solubilization, the rate of solubilization and pellet nitrogen (a proxy for cell concentration) for the four end-strains of the strain lineages 1 and 2. Each of the four strains was able to emulate the extent of solubilization for wild-type when cultivated on loadings of 120 g gluc eq/L, the highest amount of cellulose was solubilized by strain LL374 at 109.2 g gluc eq/L. Since the ATP yield per sugar is limited in anaerobes, and production of cellulosomes is energetically expensive, reducing ATP supply by deleting *pta* could result in decreased cellulosome production, which would have a negative effect on the rate of cellulose solubilization. Two of the strains tested (LL374 and LL1043) had lower maximum solubilization rates, but remained close to the wild-type solubilization rate. The rate value for strain LL375 was within the range of wild-type, while strain LL1011 surpassed the maximum rate of solubilization at 3.19 g gluc eq/L/h compared to wild-type *C. thermocellum.*

The maximum cell concentration [cell]_max_ for wild-type *C. thermocellum* grown on 103.4 g gluc eq/L is documented at 0.64 g N/L [13]. None of the four strains tested here exceeded that value, (0.47–0.56 gN/L). One possible reason for this lower cell concentration is the deletion of *pta*, which results in a loss of ATP. In the chemostat experiments strains with *pta* knocked out had a lower steady-state cell concentration. However, it is important to note that the lower amount of cells (biocatalyst) did not diminish or negatively affect the extent of solubilization. Just as with wild-type, for all strains the maximum rate of cellulose solubilization happens before peak cell growth (shown in the left panel set of figures and indicated by the green (solubilization rate) and red (cells) arrows in the middle and right panels of Fig. 7). More extensive metabolic engineering than described here by knocking out genes involved in acetate, lactate, formate and hydrogen production affected the growth rate of the newly generated strain, and was only partially recovered after extensive evolutionary engineering involving serial transfers and selection & screening based ALE approach [32, 33]. To avoid negative effects on cell physiology and growth performance current metabolic engineering strategies involve introducing heterologous genes for ethanol-producing pathway [11, 29, 33, 34]; Hon et al. [15], describe expressing four genes from *Thermoanaerobacterium saccharolyticum* in *C. thermocellum* that increased ethanol titer and yield while not negatively affecting growth rate or the ability to solubilize cellulose.

Under similar high solids cultivation conditions as described here, wild-type *C. thermocellum* makes 13.9 g/L ethanol, 11.3 g/L acetate, 2.6 g/L formate, 0.8 g/L fumarate and 0.8 g/L lactate [13]. While both the acetate and lactate pathways are disrupted for all strains in the high cellulose fermentation experiments, there were non-negligible amounts of acetate (1.2–3.1 g/L) and lactate (1.3–1.8 g/L) detected (see Additional file 11: Table S11). As mentioned earlier, this is likely a result of side product formation from other biological reactions. Formate reaches concentrations between 0.7 and 1.0 g/L, which is an actual decrease compared to wild-type. There is a notable increase in extracellular fumarate for the end-strains from lineage 2; LL374 (1.2 g/L) and LL375 (2.8 g/L) while strains from lineage 1 are similar to wild-type (0.6 g/L for LL1011 and 0.9 g/L for LL1043).

Higher alcohols as well as extracellular amino acids are undesired fermentation products in a for-ethanol engineering scenario. Wild-type *C. thermocellum* produces 1.6 g/L isobutanol, 0.42 g/L 2,3-butanediol, and total amino acids were determined at 7.5 g/L (with valine at 4.5 g/L and alanine at 1.5 g/L) and pyroglutamate at 0.2 g/L. The pyroglutamate detected here is believed to be the result of a degradation reaction of glutamate and of glutamine at increased temperatures based on [35, 36]. Both isobutanol and free amino acids (including pyroglutamate) remain a major side product in engineering-for-ethanol followed by ALE (Fig. 7 the middle and right panels); with *ldh* and *pta* knocked out, valine becomes the second most abundant fermentation product with a maximum concentration of 7.0 g/L for stain LL375 and a minimum concentration of 4.9 g/L for strain LL1043. To reduce the superfluous production and excretion of amino acids, one option is to knock out parts of nitrogen metabolism. Rydzak et al. have approached this by deleting a type I glutamate synthetase *glnA* which resulted in lower amino acid production and a higher ethanol yield [37]. For the chemostat experiments the free amino acids concentration and extracellular pyruvate went down after ALE. Under high cellulose conditions pyruvate is present for all four strains (0.9–1.3 g/L see Additional file 11: Table S11), the maximum concentration pyruvate documented for wild-type *C. thermocellum* is 0.3 g/L.

Strains LL1011 and LL1043 from lineage 1 produced more isobutanol than strains LL374 and LL375 from lineage 2, which produced more than wild-type. However, the two strains from lineage 2 produced more 2,3-butanediol than the strains from lineage 1, which produced more than wild-type. It remains unclear if this is due to the presence or absence of the *AdhE*^D494G^ mutation. While we do not know of reported attempts of engineering *C. thermocellum* for butanediols, efforts have been made to engineer *C. thermocellum* into producing only isobutanol with plasmid-based heterologous gene expression using genes from *Geobacillus stearothermophilus* and *Lactococcus lactis* [38]. *C. thermocellum*’s native ability to produce isobutanol, which is believed to involve both ketoisovalerate ferredoxin-dependent reductase (KOR) activity and pyruvate ferredoxin oxidoreductase (PFOR) activity [38, 39], has not been subject to engineering. However, Hon et al. describe one of the side effects of knocking out a specific *pfor* gene which eliminated native isobutanol production, this would have the potential to redirect metabolism towards ethanol. The maximum isobutanol titer detected in this set of experiments was 5.1 g/L by strain LL1043, which is close to the maximum titer reported for a for-isobutanol-engineered strain reaching a value of 5.4 g/L at a yield of 48% [38]. Note that the results for the isobutanol engineered strain were obtained under different cultivation conditions.

Finally, the ethanol titer was significantly higher for three out of the four strains compared to wild-type. LL1043 had the highest ethanol titer reported to date at 29.9 g/L. Although it seems that the *AdhE*^D494G^ mutation caused the ethanol titer for both strains in lineage 1 to be higher, as with the results from the chemostat experiments, LL374 also has a very high ethanol titer at 24.5 g/L. For high cellulose loadings LL374 actually has a higher ethanol titer compared to LL1011 (with the *adhE*^D494G^ mutation). LL1043 has both the highest ethanol titer and isobutanol titer, the lowest free amino acids concentration and the lowest [cell]_max_ from all four strains, while it went through the second highest amount of solubilized cellulose. In the chemostat experiments the absolute difference in ethanol titer between strains LL374, LL1011 and LL1043 was minimal, but there was no nominal improvement for strain LL375 after ALE. Under high cellulose concentration strain LL375 still produces high concentrations of amino acids and pyruvate (see Additional file 11: Table S11) and only a small improvement in ethanol titer compared to wild-type. Notwithstanding the extensive analysis described in this paper, we did not gain additional insight into why ALE improved the growth rate but not the ethanol titer for this strain. Carbon recoveries for the cellulose fermentations are listed in Additional file 11: Table S11, the highest recovery was for strain LL1043 at 94.2%, with strains LL374 (78.5%) and LL1011 (77.9%) around similar values as described for wild-type (80.8%) [13] and only slightly lower for strain LL375 (71.5%).

Metabolic engineering has been applied to cellulolytic microorganisms as genetic tools have become available, and in a few cases has involved ALE. Guedon et al. [40] heterologously expressed pyruvate decarboxylase and alcohol dehydrogenase II from *Zymomonas mobilis* in the mesophile *Clostridium cellulolyticum* increasing biomass concentration and cellulose consumption as well as a 50% increase in ethanol titer. In 2012 Li et al. inactivated lactate and malate dehydrogenase in *C. cellulolyticum* resulting in a titer of 2.7 g/L ethanol from cellulose [41]. Tolonen et al. increased the ethanol tolerance of *Clostridium phytofermentans* by serial transfer and followed an approach similar to Guedon et al., leading to an ethanol titer of around 0.9 g/L [42]. Chung et al. engineered the hyperthermophile *Caldicellulosiruptor bescii* to produce 0.6 g/L ethanol from switchgrass by heterologously expressing *adhE* from *C. thermocellum* [43]. Metabolic engineering studies related to cellulolytic organisms other than *C. thermocellum* have often targeted higher alcohols. These include production of isobutanol in the mesophilic *C. cellulolyticum* via heterologous expression of genes from other mesophiles [44–46]. *Clostridium cellulovorans*, also a mesophile, has been engineered to produce n-butanol via a combination of heterologous expression of genes from *Clostridium acetobutylicum* and ALE [47–50].

It is instructive to consider microorganisms to which metabolic engineering, ALE, and genomics-enabled approaches have been applied more extensively than is the case for *C. thermocellum* and other cellulolytic anaerobes. *Corynebacterium glutamicum* is case in point [51]. Becker et al. combined metabolic modeling, ^13^C flux analysis and metabolic engineering to create a lysine-overproducing *C. glutamicum* strain with lysine production up to 120 g/L [52], competitive with classically derived strains. Mahr et al. used a combination of iterative cultivation and fluorescence-based cell sorting to increase both growth rate and product formation while reducing by-product formation [53]. Lee et al. used a combination of metabolic engineering and adaptive evolution to enable a *C. glutamicum* strain to utilize cellobiose and xylose [54]. Radek et al. applied a combination of automated ALE in microplates based on serial transfer of a xylose utilizing strain of *C. glutamicum* increasing its growth rate 2.6-fold [55]. Zhang et al. engineered *C. glutamicum* by gene inactivation, gene knock-out and overexpression towards a l-ornithine titer of 18.7 g/L [56]. Wen and Bao [57] used pathway engineering targeting metabolic control to produce 62.5 g/L lysine from corn stover hydrolysate. *C. glutamicum* has been developed to the point that it may plausibly be considered as an alternative to *E. coli* as a platform for products other than amino acids [58, 59].

Looking toward the future, improved production of ethanol, and eventually other products, by *C. thermocellum* is reasonable to anticipate in light of what has been accomplished with more extensively studied microorganisms. Although ethanol production exceeding 30 g/L has yet to be reported for *C. thermocellum*, the organism can grow on both cellulose and cellobiose in the presence of 50 g/L ethanol following ALE via serial transfer [24]. Experience with other microorganisms suggests that the difference between maximum product concentrations tolerated and produced can be closed with sufficient effort [60], with propanediol in *E. coli* being a prominent example. The example of the hemicellulose-fermenting *T. saccharolyticum*, which has been engineered to produce > 60 g/L ethanol [61], further supports the potential for developing *C. thermocellum* into an industrial microorganism.

## Conclusions

In contrast to knocking out *ldh*, which has minimal influence on fermentation end-products, deleting *pta* causes a shift from acetate production to pyruvate and amino acids and a decrease in biomass (including growth rate) while not increasing ethanol production. After ALE (adaptive laboratory evolution), metabolism for three out of four strains with *ldh* and *pta* knocked-out shifts from extracellular pyruvate and free amino acids to ethanol.

The distribution of fermentation products is generally not regulated at the transcript level (with one minor exception for valine in strain LL1042).

The *adhE*^D494G^ mutation is important for ethanol production. Re-introducing this mutation into a strain with a wild-type *pta* locus resulted in an ethanol yield of 45% of the theoretical maximum, compared to ~ 50% for the evolved *ldh pta* deletion strains on 5 g/L cellobiose. At higher substrate loadings, we observed higher levels of ethanol production in strain LL1043 compared to strain LL1011. Since both strains contain the *adhE*^D494G^ mutation, the phenotype must result from one of the other mutations. Strain LL374 showed increased ethanol production, but does not have the *adhE*^D494G^ mutation (or any other mutations in *adhE*). In that strain, the most likely candidate is a frameshift mutation in the *hfsB* hydrogenase gene, however confirmation of this hypothesis awaits further experimental data.

The flux split between ethanol and acetate is likely controlled by availability of electrons, not carbon. Evidence supporting this hypothesis is that pyruvate accumulates initially upon deletion of *pta* (and presumably acetyl-CoA accumulates as well), but ethanol production is unchanged. By contrast, introducing the *adhE*^D494G^ mutation immediately increases ethanol production. The *adhE*^D494G^ mutation is known to expand cofactor specificity of ADH reaction from NADH-only to either NADH or NADPH [28] thus the mutant *adhE* has access to both the NADH and NADPH pools, where previously it only had access to the NADH pool.

Finally, when cultured under industrially relevant levels of cellulose, strains that have undergone ALE can solubilize up to 109.2 g glucose equivalent/L cellulose, which represent an undiminished cellulolytic ability compared to wild-type *C. thermocellum*. Knocking out pathways for lactate and acetate make valine the second fermentation product after ethanol. An ALE-generated *ldh*-*pta* knock-out strain of *C. thermocellum* with the *AdhE*^D4949G^ mutation produced 29.9 g/L ethanol and 5.1 g/L isobutanol. This supports the hypothesis that a strain of *C. thermocellum* can be developed able to produce ethanol at high yield and still rapidly solubilize and utilize cellulose.

## Methods

### Microbial strains

Strains used in this study are listed in Table 2; strain lineages are also presented diagrammatically in “Results and discussion” section.

**Table 2:** Strains used in this study

Strain ID	Parent strain ID	Alternate designation	Genotype	Sequence data accession number	References
LL345	LL1004	M1354	∆*hpt*	SRP053786	[8]
LL372	LL376	M1629	∆*hpt* ∆*spo0A* ∆*ldh*	SRP083692	[9]
LL373	LL376	M1630	∆*hpt* ∆*spo0A* ∆*pta*	SRP083691	[9]
LL374	LL1044	M1724	∆*hpt* ∆*spo0A* ∆*ldh* ∆*pta*::*PgapD*-*cat*-*hpt* adapted by pH auxostat	SRP083690	[62]
LL375	LL1044	M1725	∆*hpt* ∆*spo0A* ∆*ldh* ∆*pta*::*PgapD*-*cat*-*hpt* adapted by chemostat	SRP083695	[9]
LL376	LL345	M1726	∆*hpt* ∆*spo0A*	SRP083697	[9]
LL1004		DSM1313	Wt	SRP077312	DSMZ culture collection
LL1011	LL1042		∆*hpt* ∆*ldh* ∆*pta* adapted by serial transfer	SRP054852	[62]
LL1036	LL345	M1407	∆*hpt* ∆*ldh*	SRP054849	[8]
LL1041	LL345	M1448	∆*hpt* ∆*pta*	SRP054855	[8]
LL1042	LL1036	M1434	∆*hpt* ∆*ldh* ∆*pta*	SRP054854	[8]
LL1043	LL1042	M1570	∆*hpt* ∆*ldh* ∆*pta*::*PgapD*-*cat*-*hpt* adapted by pH auxostat	SRP053784	[8]
LL1044	LL372	M1655	∆*hpt* ∆*spo0A* ∆*ldh* ∆*pta*::*PgapD*-*cat*-*hpt*	SRP077297	[9]
LL1111	LL345		∆*hptldh*^S161R^ ∆*adhE*	SRP049310	[11]
LL1160	LL1111		∆*hptldh*^S161R^ ∆*adhE*::*adhE*	SRP059563	[28]
LL1161	LL1111		∆*hptldh*^S161R^ ∆*adhE*::*adhE*^D494G^	SRP059562	[28]

Methodological details for the development of strains via ALE are as follows: wild-type *C. thermocellum* strain DSM 1313 was originally obtained from the DSMZ culture collection. Strain LL374 was adapted from LL1044 by culturing in a pH auxostat for 500 h. Strain LL375 was adapted from LL1044 by culturing in a chemostat for 500 h. Strain LL1011 was adapted from LL1042 by culturing in a pH auxostat. Strain LL1043 was adapted from LL1042 by 60 serial transfers with 1:100 dilution (approximately 200 generations). All strains in Table 2 except LL1111, LL1160 and LL1161 were originally developed by Mascoma Corporation. Strain construction is described in detail in the references in Table 2. All strains were conserved in the Lynd laboratory strain collection cultured on CTFUD [22] in 1 mL aliquots stored at − 80 °C. For use in the experiments described here, freezer stocks of all strains were made on MTC medium (see “Growth media”). For each strain an overnight 50 mL working volume serum bottle culture was aliquoted into 5 mL volumes and stored at − 80 °C.

### Growth media

Medium for thermophilic clostridia (MTC) was used for making freezer strain stocks and for cultivation on high cellulose loadings [13], and modified low-carbon medium (LCmed) used for microplate growth rate assays and carbon-limited chemostat cultivation [31].

For generating freezer stocks MTC contained 5 g/L crystalline cellulose (Avicel^®^ PH105) as carbon source, 5 g/L 3-*N*-morpholino propanesulfonic acid (MOPS) for pH buffering and 2 g/L urea (CH_4_N_2_O) as nitrogen source; final concentrations: 5 g/L cellulose ([C_6_H_10_O_5_]_x_), 2.0 g/L citric acid tripotassium salt (C_6_H_5_O_7_K_3_), 1.25 g/L citric acid monohydrate (C_6_H_5_O_7_·H_2_O), 1.0 g/L Na_2_SO_4_, 1.0 g/L KH_2_PO_4_, 2.5 g/L NaHCO_3_, 1.0 g/L MgCl_2_·6H_2_O, 0.2 g/L CaCl_2_·2H_2_O, 0.1 g/L FeCl_2_·4H_2_O, 1.0 g/L l-cysteine HCl·H_2_O, 20 mg/L pyridoxamine dihydrochloride, 4 mg/L PABA, 2 mg/L d-biotin, 2 mg/L B_12_, 6 mg/L MnCl_2_·4H_2_O, 2.5 mg/L ZnCl_2_, 0.6 mg/L CoCl_2_·6H_2_O, 0.6 mg/L NiCl_2_·6H_2_O, 0.6 mg/L CuSO_4_·5H_2_O, 0.6 mg/L H_3_BO_3_ and 0.6 mg/L Na_2_MoO_4_·2H_2_O.

For high cellulose loading cultivation in bioreactors MTC contained 120 g/L crystalline cellulose as carbon source, no MOPS and 5 g/L urea as nitrogen source. Vitamins and trace elements were increased with final concentrations at 80 mg/L pyridoxamine dihydrochloride, 16.0 mg/L para-aminobenzoic acid (PABA), 8 mg/L d-biotin and 18 mg/L vitamin B_12_, 5.0 mg/L MnCl_2_·4H_2_O, 2.5 mg/L ZnCl_2_·6H_2_O, 0.5 mg/L CoCl_2_·6H_2_O, 0.5 mg/L NiCl_2_·6H_2_O, 0.5 mg/L CuSO_4_·5H_2_O, 0.5 mg/L H_3_BO_3_ and 0.5 mg/L Na_2_MoO_4_).

For cultivation in chemostats modified LCmed contained 5 g/L cellobiose (C_12_H_22_O_11_), 2.0 g/L KH_2_PO_4_, 3.0 g/L K_2_HPO_4_, 0.1 g/L Na_2_SO_4_, 0.5 g/L urea (CH_4_N_2_O), 0.2 g/L MgCl_2_·6H_2_O, 0.05 g/L CaCl_2_·2H_2_O, 0.0035 g/L FeSO_2_·7H_2_O, 0.025 g/L FeCl_2_·4H_2_O, 1.0 g/L l-cysteine HCl·H_2_O, 20 mg/L pyridoxamine dihydrochloride, 4 mg/L PABA, 2 mg/L d-biotin, 2 mg/L B_12_, 6 mg/L MnCl_2_·4H_2_O, 2.5 mg/L ZnCl_2_, 0.6 mg/L CoCl_2_·6H_2_O, 0.6 mg/L NiCl_2_·6H_2_O, 0.6 mg/L CuSO_4_·5H_2_O, 0.6 mg/L H_3_BO_3_ and 0.6 mg/L Na_2_MoO_4_·2H_2_O. This medium was also used for the plate reader assays, but then complemented with 5 g/L MOPS for pH buffering.

All media were prepared in 6 (bioreactor experiments) or 7 (bottle and plate reader experiments) separate solutions as indicated in Holwerda et al. [31].

### Growth rate analysis

Growth rate and culture density (optical density at 600 nm, i.e., OD_600_) were determined by measuring the OD_600_ every 3 min for cultures grown in a covered 96-well plate placed in a plate reader (BioTek, Winooski, VT). The plate reader was modified by the manufacturer to enable cultivation at 55 °C, and measurements were based on ≥ triplicate cultivations on modified LC medium containing 5 g/L cellobiose. The plate was automatically briefly shaken before recording every data point. All strains were cultured on modified LC medium before being used as inoculum. BioTek Gen5 microplate reader data analysis software enabled rate determination in the logarithmic range of growth.

### Fermentation products, cell biomass, amino acids and cellulose solubilization measurements

The fermentation products acetate, lactate, formate, ethanol, 2,3-butanediol, isobutanol, fumarate, pyruvate, malate, pyroglutamate and the sugars glucose and cellobiose were measured by high performance liquid chromatography (HPLC, Waters, Milford, MA) using both refractive index and UV at 210 and 250 nm with an Aminex HPC-87H column (Bio-Rad Hercules, CA) as described in [13]. Pellet carbon and nitrogen as proxy for cellular biomass were measured on a Shimadzu TOC-Vcph total organic carbon analyzer with an additional total nitrogen unit (Shimadzu Scientific Instruments, Columbia MD). An acidified glycine solution was used as a standard for both carbon and nitrogen [63]. Residual cellulose for the high cellulose batch cultivations was determined by quantitative saccharification [64] as described in [13].

### Amino acids determination

Free amino acids (alanine, arginine, asparagine, aspartic acid, glutamic acid, glutamine, histidine, isoleucine, leucine, lysine, methionine, phenylalanine, proline, serine, threonine, tryptophan, tyrosine, and valine) were determined from broth supernatant by mass spectrometry against a ^15^N labeled amino acid standard (Cell Free Amino Acids mix, Cambridge Isotope Laboratories, Tewksbury, MA), which were in turn quantified against purified non-labeled amino acids (Sigma-Aldrich, St. Louis, Mo). Samples were mixed with ^15^N-labeled standards in varying ratios to give roughly equivalent final MS intensities for each amino acid (within 1/10 of each other). *C. thermocellum* broth supernatant (culture broth centrifuged at 21,130×*g*/15,000 rpm for 2–5 min and pellet removed) was diluted 1 in 10 in HPLC running buffer and analyzed by HPLC/MS. HPLC/MS conditions were as described in Tian et al. [65], generally injecting 10-µl samples. Results were corrected for natural abundance of ^15^N.

### Anthrone assay for residual supernatant carbohydrates

Soluble carbohydrate present in the supernatant was quantified using the anthrone assay. Anthrone reagent (anthrone, water, sulfuric acid and ethanol) was added to supernatant aliquots in triplicates, mixed and heated to 100 °C for 20 min. After color development, carbohydrates were measured versus a serial dilution of a glucose standard at 650 nm [66]. Glucose and cellobiose concentrations measured prior by HPLC were subtracted from the total to eliminate double counting.

### Hydrogen gas analysis

The headspace of the chemostats was continuously purged with nitrogen gas at 5 mL/min, the off-gas was channeled through a detachable 150-mL serum bottle stoppered with a butyl rubber stopper. At the time of sampling this bottle was disconnected from the off-gas stream and saved for gas analysis. The hydrogen gas concentration in the serum bottle was measured on a 310 Educational gas chromatograph (SRI Instruments, Torrence CA) with a HayeSep D packed column (151 °C) and thermal conductivity detector against know standards with nitrogen as carrier gas (8.2 mL/min). PeakSimple software (SRI Instruments, Torrence CA) aided in the determination of the hydrogen concentration in the off-gas.

### Bioreactor set-up and cultivation conditions

The chemostat bioreactor set-up consisted of 0.5 L custom-built glass fermentation vessels (NDS Technologies, Vineland NJ) controlled by a Qplus multiplex cultivation system (Sartorius Stedim, Bohemia NY) with a working volume of 300 mL, maintained at 55 °C by a 19.6 L Polystat external water bath (Cole Parmer, Vernon Hills Il). The headspace was continuously flushed with ‘Ultrapure’ nitrogen gas (Airgas, White River Junction VT) at 5 mL/min to ensure a slight overpressure in the vessel headspace, the condenser was kept at 4–5 °C with a 6L Polystat cooling bath (Cole Parmer, Vernon hills Il). Medium was supplied by continuous operation of a Watson-Marlow 205S peristaltic pump (Watson-Marlow, Wilmington MA). The effluent pump was automatically activated by level control; the level of the fermentation broth closed an electrical circuit, which activated the pump. The feed carboy and effluent vessels were placed on scales with accompanying software (A&D company, San Jose, CA), data were collected every minute from which the dilution rate could be calculated with high accuracy. The pH level of the bioreactor was maintained at 7.0 by a gel-filled pH probe (Mettler-Toledo, Billerica, MA) and automated addition of 4 N KOH. Optical density in the near-infrared range (NIR) was monitored in situ by a DASGIP OD4 system at 850 nm (Dasgip Eppendorf, Hauppauge, NY) for each chemostat run which enabled closely monitoring of cellular biomass dynamics and determination of steady state. Each strain, except LL1111, was cultivated in duplicate in chemostats and each individual chemostat run was sampled twice; the first time after at least 4 residence times (≥ 40 h) and a second time after at least 7 residence times (≥ 70 h) from initiation of the continuous feed mode. The feed was started after culturing for ~ 20 h in batch mode and was only initiated after the OD_850_ had reached a maximum. All strains (except LL1111) were grown in chemostats under carbon-limiting conditions at a dilution rate of 0.1 h^−1^.

High cellulose batch fermentations were carried out as described previously [13], but with 120 g/L and without intermittent addition of vitamins. Cultivation conditions for high solids reactors were the same as with the chemostat cultivation, except the stirring rate was set 300 rpm, the working volume was 1 L and the medium used was MTC. Fermentations were sampled for at least 12 time points, and were stopped 220 h after inoculation by which point base addition and gas formation had ceased. Each of the end-strains tested at high solids was cultivated in duplicate. The rate of substrate solubilization was determined by fitting a sigmoidal curve to the average residual cellulose concentration of duplicate reactor runs as determined by quantitative saccharafication (QS) in gram glucose equivalents/L, each time point for each reactor run was assayed by QS in triplicate. The first derivate of the sigmoidal fit describes the rate of cellulose solubilization in gram glucose equivalents/L/h; see also [13, 67].

### Genome resequencing

Whole-genome sequencing was used to verify strain construction and check for secondary mutations. Raw data are available from the NCBI Sequence Read Archive (see accession numbers in Table 2). DNA was submitted to the Joint Genome Institute (JGI) for sequencing with an Illumina MiSeq, HiSeq 2000 or HiSeq 2500 instrument. Unamplified libraries were generated using a modified version of Illumina’s standard protocol. 100 ng of DNA was sheared to 500 bp (or in some cases 4000 bp) using a focused ultrasonicator (Covaris). The sheared DNA fragments were size-selected using SPRI beads (Beckman Coulter). The selected fragments were then end-repaired, A-tailed, and ligated to Illumina compatible adapters (IDT, Inc.) using KAPA—Illumina library creation kit (KAPA biosystems). Libraries were quantified using KAPA Biosystems’ next-generation sequencing library qPCR kit and run on a Roche LightCycler 480 real-time PCR instrument. The quantified libraries were then multiplexed into pools for sequencing. The pools were loaded and sequenced using the appropriate Illumina Reagent Kit for a 2 × 100 or 2 × 150 bp indexed run to generate paired-end reads. At the beginning, we collected 100-bp reads. When the Illumina sequencing technology improved, we switched to 150-bp reads. An insert length of 500 bp was used for routine sequencing. For some strains, we also used an insert length of 4000 bp to allow repeat regions to be resolved.

Data were analyzed with CLC Genomics Workbench v10 (Qiagen, Germantown MD). First, sequences were trimmed for quality (> 0.001) and ambiguous residues (max of 1). Then 2,500,000 reads were sampled to give an average read depth of about 105 (for 100 bp reads, 3,750,000 reads were sampled to maintain the same average read depth). Reads were mapped to the NC_017304.1 reference genome, using the default parameters, except that similarity fraction was increased from 0.8 to 0.95 to reduce mapping errors. The preliminary alignment was improved by 2 passes of local re-alignment. Mutations were identified using the “basic variant detection” and “structural variant” tools. Mutations were filtered against control reads from wt *C. thermocellum* (LL1004) to eliminate false-positive mutations due to differences between our laboratory strain of *C. thermocellum* and the reference genome.

### Mutation annotation

Mutations were classified as “origin mutations” if the mutation was not present in the parent strain (i.e., inherited). For the purposes of correlating mutations with phenotypes, inherited mutations were ignored. To identify mutations in the regulatory region of a gene, we chose an arbitrary threshold of 500 bp upstream of the target gene. A mutation was considered an ‘upstream mutation’ if it was within 500 bp upstream of the target gene and not part of another coding region.

### Gene expression analysis

#### RNA preparation

Samples for RNA were harvested from chemostats by spinning down duplicate 20 mL samples for 5 min at 3500×*g* at 5 °C in a fixed angle refrigerated centrifuge immediately after sampling (Biofuge 15R, Heraeus, Germany). Supernatant was discarded, and pellets were flash frozen in liquid nitrogen and stored at − 80 °C upon RNA isolation. Upon extraction the pellet was treated with 20 mL of RNA Protect Bacteria Reagent (Qiagen). RNA was extracted by RNeasy mini kit (Qiagen) and contaminated DNA was removed using RNase-Free DNase set (Qiagen). The resulting RNA was quantified using a Qubit 2.0 fluorometer using Life Technologies Quant-iT™ RNA Assay Kit. The standard curve contained three points (50 ng/µL, 400 ng/µL, and 1000 ng/µL) and a blank. The quality was analyzed using the Agilent 2100 BioAnalyzer. RNA purity was determined with a NanoDrop spectrophotometer, by measuring the ratio of absorbance at 260 nm vs. 280 nm.

#### RNAseq analysis

For RNAseq analysis, RNA (prepared as described in the “RNA preparation” section) was sent to the Joint Genome Institute (Walnut Creek, CA) for conversion to cDNA and Illumina sequencing. Ribosomal RNA (rRNA) was removed from 100 ng of total RNA using Ribo-Zero (TM) rRNA Removal Kit (Epicentre). Stranded cDNA libraries were generated using the Illumina Truseq Stranded RNA LT kit. The rRNA depleted RNA was fragmented (500 bp approximate size) and reversed transcribed using random hexamers and SSII (Invitrogen) followed by second strand synthesis. The fragmented cDNA was treated with end-pair, A-tailing, adapter ligation, and 10 cycles of PCR. The prepared library was quantified using KAPA Biosystem’s next-generation sequencing library qPCR kit and run on a Roche LightCycler 480 real-time PCR instrument. The quantified library was then pooled with other libraries and sequenced (see “Genome resequencing” section for details). For strains LL376, LL1044 and LL1042, one RNA prep was rejected due to low quality and no RNAseq data was generated for that sample, resulting in 3 rather than 4 replicates for those strains.

Data was normalized as follows: raw fastq file reads were filtered and trimmed using the JGI QC pipeline resulting in the filtered fastq file (*.anqrptk.fastq.gz files). Using BBDuk (http://jgi.doe.gov/data-and-tools/bbtools/bb-tools-user-guide/bbduk-guide/), raw reads were evaluated for artifact sequence by kmer matching (kmer = 25), allowing 1 mismatch and detected artifact was trimmed from the 3′ end of the reads. RNA spike-in reads, PhiX reads and reads containing any Ns were removed. Quality trimming was performed using the phred trimming method set at Q10. Following trimming, reads under the minimum length threshold of 45 bases were removed. Reads were mapped to the reference *C. thermocellum* genome (NC_017304.1). Gene expression was determined by normalizing the total number of reads for each library and then normalizing for gene length to get reads per kilobase per million mapped reads (RPKM). These data are presented in Additional files 7, 8 and 9: Table S7, Table S8 and Table S9.

Libraries BCHGZ (strain LL374) and BCGYG (LL372) showed a compressed dynamic range, which likely indicates problems with the library construction or sample processing, and these libraries were eliminated from the data. Thus, these strains have 3 rather than 4 replicates of expression data.

## Supplementary information

**Additional file 1. Table S1:** (A) Overview of cell biomass concentration, main fermentation products, hydrogen production, the cellobiose concentration in feed and the average dilution rate for each of the 15 strains. (B) Overview of all amino acids produced during fermentation for the 15 strains in milligram/Liter.

** Additional file 2: Table S2.** All mutations in all strains. Genes identified by *Clostridium thermocellum* locus number from Genbank NC_017304.1, which is also used for chromosomal coordinates of the start region of the mutation (“StartReg” column). For each mutation, the fraction of reads that support the mutation is given.

**Additional file 3: Figure S3.** Evidence for merodiploid arrangement of *pta* locus.

**Additional file 4: Figure S4.** Evidence for wild type arrangement of *gapDH* locus.

**Additional file 5: Figure S5.** Total number of mutations in each strain, identified by either DNA or RNA sequencing.

** Additional file 6: Supplemental text S6.** Discussion of convergent mutations not related to ethanol production.

**Additional file 7: Table S7.** JGI libraries. Table describing each chemostat where samples were sent for RNAseq analysis. The ID column can be used to match RNAseq data to fermentation data. The JGI library column can be used to match individual RNAseq data.

**Additional file 8: Table S8.** RNAseq expression data for each gene and library. Units of reads per kilobase per million mapped reads (RPKM). Genes are identified by *Clostridium thermocellum* locus number, from Genbank NC_017304.1. The linkage between libraries and strains is given in Additional file 7: Table S7.

**Additional file 9: Table S9.** RNAseq expression data for each gene and strain. Units of reads per kilobase per million mapped reads (RPKM), median value for each strain (n ≥ 4). Genes are identified by *Clostridium thermocellum* locus number, from Genbank NC_017304.1 and NCBI GeneInfo identifier number. Strains are identified by LL number (Table 2).

**Additional file 10: Figure S10.** Differential gene expression.

**Additional file 11: Table S11**. Table S11 (A) Overview of the residual cellulose concentration, the cell biomass concentration, main fermentation products, and amino acids produced by the four end-strains (LL374, LL375, LL1011 and LL1043) cultivated on 120 g/L cellulose (Figure 7). (B) Overview of all amino acids produced during fermentation the four end-strains on 120 g/L cellulose (Figure 7). (C) Carbon recovery for the four end-strains on 120 g/L cellulose (Figure 7).

## Data Availability

All data generated or analyzed during this study are included in this published article and its additional files. DNA sequences and resequencing results are available from GenBank via their accession numbers.

## Abbreviations

ALE: Adaptive laboratory evolution
pta: Phosphotransacetylase
adhE: Alcohol and aldehyde dehydrogenase E
hfsB: Hydrogenase-Fe-S B
ldh: Lactate dehydrogenase
8AZH: 8-Azahypoxanthine
D: Dilution rate (h^−1^)
FBP: Fructose 1,6-bisphosphate
ack: Acetate kinase
