# Supplementary material for: Metabolic and evolutionary responses of Clostridium thermocellum to genetic interventions aimed at improving ethanol production
Source: Biotechnol Biofuels. 2020 Mar 10;13:40. doi: 10.1186/s13068-020-01680-5 (PMC7063780; doi:10.1186/s13068-020-01680-5)
Supplement: Supplementary file 1 — Additional file 1. Table S1: (A) Overview of cell biomass concentration, main fermentation products, hydrogen production, the cellobiose concentration in feed and the average dilution rate for each of the 15 strains. (B) Overview of all amino acids produced during fermentation for the 15 strains in milligram/Liter. [file 13068_2020_1680_MOESM1_ESM.pdf]

Table S1: (A) Overview of cell biomass concentration, main fermentation products, hydrogen production, the cellobiose concentration in feed and the average dilution rate for each of the 15 strains.

*Hydrogen values are calculated compared to wild-type and shown as a relative value versus the wild-type data arbitrarily set at 1.

Strain	Total number of replicates	Pellet C (gC/L)	Pellet N (gN/L)	Acetate (g/L)	Ethanol (g/L)	Formate (g/L)	Lactate (g/L)	Pyruvate (g/L)	Total AA's (g/L)	H_2 in headspace*	Cellobiose in feed (g/L)	Dilution rate ($hour^{-1}$)
LL1004<br>wild-type	12	0.328<br>±0.019	0.098<br>±0.005	0.925<br>±0.024	0.840<br>±0.016	0.206<br>±0.014	0.0212<br>±0.0025	0.012<br>±0.001	0.068<br>±0.008	1.000	4.851<br>±0.141	0.097<br>±0.007
LL345<br>Δhpt	4	0.354<br>±0.016	0.104<br>±0.000	0.979<br>±0.004	0.711<br>±0.010	0.265<br>±0.020	0.0179<br>±0.0014	0.012<br>±0.002	0.066<br>±0.005	1.053	4.689<br>±0.03	0.098<br>±0.004
LL1041<br>Δhpt Δpta	4	0.260<br>±0.011	0.072<br>±0.002	0.033<br>±0.003	0.848<br>±0.028	0.055<br>±0.006	0.0898<br>±0.0032	1.151<br>±0.053	0.341<br>±0.015	0.637	4.750<br>±0.602	0.010<br>±0.002
LL1036<br>Δhpt Δldh	4	0.348<br>±0.011	0.102<br>±0.003	0.960<br>±0.02	0.766<br>±0.025	0.285<br>±0.02	0.0132<br>±0.0005	0.0000<br>±0	0.068<br>±0.005	1.017	4.868<br>±0.140	0.109<br>±0.001
LL1042<br>Δhpt Δldh Δpta	4	0.256<br>±0.020	0.072<br>±0.005	0.025<br>±0.002	0.876<br>±0.025	0.149<br>±0.024	0.0516<br>±0.003	0.893<br>±0.107	0.404<br>±0.082	0.475	4.747<br>±0.101	0.109<br>±0.001
LL1011<br>Δhpt Δldh Δpta adapted	4	0.209<br>±0.009	0.057<br>±0.002	0.021<br>±0.001	1.350<br>±0.038	0.183<br>±0.005	0.0095<br>±0.0018	0.227<br>±0.016	0.272<br>±0.067	0.248	4.881<br>±0.070	0.095<br>±0.002
LL1043<br>Δhpt Δldh Δpta::PgapD-cat-hpt adapted	4	0.256<br>±0.005	0.072<br>±0.002	0.019<br>±0.000	1.316<br>±0.046	0.147<br>±0.006	0.0104<br>±0.0003	0.323<br>±0.026	0.244<br>±0.034	0.255	4.850<br>±0.041	0.092<br>±0.002
LL376<br>Δhpt Δspo0A	4	0.363<br>±0.010	0.105<br>±0.003	1.030<br>±0.011	0.641<br>±0.043	0.328<br>±0.025	0.0176<br>±0.0047	0.013<br>±0.002	0.065<br>±0.010	0.994	4.689<br>±0.031	0.102<br>±0.001
LL373<br>Δhpt Δspo0A Δpta	4	0.252<br>±0.014	0.067<br>±0.003	0.031<br>±0.003	0.856<br>±0.028	0.064<br>±0.013	0.1427<br>±0.0631	0.999<br>±0.213	0.342<br>±0.029	0.582	4.750<br>±0.602	0.099<br>±0.003
LL372<br>Δhpt Δspo0A Δldh	4	0.359<br>±0.007	0.107<br>±0.005	1.0480<br>±0.013	0.666<br>±0.039	0.317<br>±0.026	0.0104<br>±0.0007	0.000<br>±0.000	0.063<br>±0.010	1.122	4.868<br>±0.140	0.102<br>±0.002
LL1044<br>Δhpt Δspo0A Δldh Δpta::PgapD-cat-hpt	4	0.233<br>±0.007	0.059<br>±0.003	0.033<br>±0.003	0.774<br>±0.011	0.123<br>±0.007	0.0514<br>±0.002	1.230<br>±0.020	0.403<br>±0.058	0.648	4.747<br>±0.101	0.100<br>±0.001
LL374<br>Δhpt Δspo0A Δldh Δpta::PgapD-cat-hpt adapted	8	0.218<br>±0.024	0.061<br>±0.010	0.024<br>±0.003	1.304<br>±0.134	0.156<br>±0.030	0.0204<br>±0.0077	0.521<br>±0.293	0.130<br>±0.053	0.246	4.784<br>±0.137	0.102<br>±0.010
LL375<br>Δhpt Δspo0A Δldh Δpta::PgapD-cat-hpt adapted	4	0.196<br>±0.019	0.048<br>±0.004	0.041<br>±0.017	0.829<br>±0.055	0.140<br>±0.015	0.0652<br>±0.0115	0.992<br>±0.076	0.436<br>±0.071	0.511	4.850<br>±0.041	0.092<br>±0.003
LL1160<br>Δhpt ΔadhE::adhE	4	0.312<br>±0.009	0.085<br>±0.003	0.774<br>±0.012	0.626<br>±0.011	0.158<br>±0.023	0.5790<br>±0.0328	0.010<br>±0.002	0.078<br>±0.004	1.057	4.689<br>±0.03	0.099<br>±0.001
LL1161<br>Δhpt ΔadhE::adhE[D494G]	4	0.296<br>±0.013	0.077<br>±0.003	0.523<br>±0.008	1.097<br>±0.078	0.240<br>±0.013	0.1317<br>±0.0156	0.008<br>±0.001	0.073<br>±0.004	0.457	4.689<br>±0.03	0.097<br>±0.000

Table S1: (B) Overview of all amino acids produced during fermentation for the 15 strains in milligram/Liter.

Strain	Total number of replicates	Alanine	Arginine	Asparagine	Aspartic acid	Glutamic acid	Glutamine	Histidine	Isoleucine	Leucine	Lysine	Methionine	Phenyl alanine	Proline	Serine	Threonine	Tryptophan	Tyrosine	Valine
LL1004	12	12.1	0.0	1.4	0.9	19.2	0.6	0.0	4.7	3.8	1.0	0.2	2.3	3.6	3.7	3.7	0.8	1.4	8.5
wild-type		±1.2	±0.0	±0.2	±0.3	±3.7	±0.3	±0.0	±0.7	±0.3	±0.6	±0.0	±0.9	±0.3	±0.5	±0.3	±0.1	±0.2	±2.1
LL345	4	11.5	0.0	0.7	0.9	20.4	0.4	0.0	4.5	3.3	0.5	0.3	2.6	3.8	3.6	3.7	0.5	1.6	7.1
Δhpt		±0.8	±0.0	±0.2	±0.2	±2.3	±0.2	±0.0	±0.7	±0.1	±0.5	±0.0	±0.7	±0.0	±0.4	±0.2	±0.1	±0.2	±0.9
LL1041	4	26.4	0.0	1.2	1.7	36.6	1.0	0.0	28.9	4.3	0.1	0.9	5.5	3.1	4.3	8.9	0.5	2.6	215.4
Δhpt Δpta		±2.1	±0.0	±0.2	±0.1	±2.7	±0.2	±0.0	±0.3	±0.1	±0.2	±0.4	±0.7	±0.4	±0.2	±0.5	±0.1	±0.3	±19.8
LL1036	4	11.8	0.0	0.7	0.8	15.2	0.7	0.0	5.3	4.3	0.4	0.1	2.8	3.0	3.0	3.4	0.7	1.6	13.8
Δhpt Δldh		±1.1	±0.0	±0.1	±0.1	±0.7	±0.3	±0.0	±0.6	±1.0	±0.4	±0.0	±0.7	±0.2	±0.1	±0.3	±0.1	±0.1	±1.9
LL1042	4	15.8	0.0	1.2	2.0	32.6	0.7	0.0	56.2	2.5	0.0	0.2	6.6	2.2	4.5	8.4	0.5	2.4	268.6
Δhpt Δldh Δpta		±0.8	±0.0	±0.3	±0.6	±6.4	±0.3	±0.0	±9.9	±2.9	±0.0	±0.1	±2.7	±0.2	±0.2	±2.2	±0.1	±0.5	±70.7
LL1011	4	14.7	0.0	1.5	2.1	33.4	0.8	0.0	12.4	5.8	0.2	0.2	3.6	2.5	4.4	6.3	0.7	1.6	153.1
Δhpt Δldh Δpta adapted		±1.1	±0.0	±0.2	±0.4	±3.7	±0.4	±0.0	±1.7	±1.1	±0.1	±0.1	±1.0	±0.2	±0.4	±0.6	±0.0	±0.1	±26.4
LL1043	4	13.1	0.0	2.1	3.7	45.8	0.5	0.0	25.8	4.5	0.4	0.2	2.7	2.1	4.2	8.3	0.6	1.7	156.2
Δhpt Δldh Δpta::PgapD-cat-hpt adapted		±1.3	±0.0	±0.3	±0.8	±9.1	±0.2	±0.0	±6.7	±0.2	±0.3	±0.1	±0.1	±0.2	±0.6	±1.4	±0.1	±0.3	±47.1
LL376	4	12.4	0.0	0.8	0.8	16.4	0.5	0.0	4.3	3.8	0.1	0.2	3.6	3.6	3.7	3.6	0.6	1.8	9.3
Δhpt Δspo0A		±2.0	±0.0	±0.2	±0.3	±3.2	±0.1	±0.0	±0.7	±0.2	±0.1	±0.1	±2.1	±0.5	±0.8	±0.6	±0.1	±0.3	±3.8
LL373	4	24.4	0.0	1.0	1.6	29.3	0.3	0.0	31.1	4.7	0.0	0.7	9.8	3.3	3.6	7.9	0.5	2.7	221.2
Δhpt Δspo0A Δpta		±4.1	±0.0	±0.2	±0.5	±5.3	±0.2	±0.0	±2.3	±0.9	±0.0	±0.4	±2.0	±0.2	±0.3	±0.8	±0.2	±0.2	±18.9
LL372	4	12.5	0.0	1.0	0.7	15.1	0.6	0.0	4.4	3.7	0.2	0.2	3.0	3.5	3.3	3.6	0.7	2.0	8.2
Δhpt Δspo0A Δldh		±1.6	±0.0	±0.1	±0.1	±2.7	±0.2	±0.0	±0.8	±0.7	±0.2	±0.0	±0.8	±0.6	±0.4	±0.5	±0.0	±0.4	±2.1
LL1044	4	17.3	0.0	1.6	2.7	48.7	0.6	0.0	41.6	1.0	0.1	6.3	4.8	4.1	5.3	11.9	0.4	2.5	253.9
Δhpt Δspo0A Δldh Δpta::PgapD-cat-hpt		±0.9	±0.0	±0.3	±0.5	±4.0	±0.4	±0.0	±6.2	±2.0	±0.1	±5.3	±0.7	±0.4	±0.2	±1.8	±0.1	±0.3	±43.2
LL374	8	15.9	0.0	0.7	0.6	16.2	0.1	0.0	10.7	3.7	0.0	0.1	11.0	2.2	1.8	3.9	0.5	3.1	59.1
Δhpt Δspo0A Δldh Δpta::PgapD-cat-hpt adapted		±3.0	±0.1	±0.2	±0.2	±2.6	±0.1	±0.1	±5.1	±0.7	±0.1	±0.0	±6.5	±0.4	±0.3	±1.2	±0.1	±1.2	±43.1
LL375	4	16.4	0.0	1.3	3.2	54.5	0.7	0.0	36.8	7.4	0.3	0.9	11.2	3.9	5.8	7.3	0.6	4.0	281.6
Δhpt Δspo0A Δldh Δpta::PgapD-cat-hpt adapted		±2.3	±0.0	±0.3	±0.9	±19.9	±0.7	±0.1	±8.8	±1.0	±0.3	±0.4	±2.6	±0.3	±0.4	±0.7	±0.1	±0.3	±46
LL1160	4	13.5	0.0	2.6	2.1	19.0	1.3	0.0	4.8	4.3	0.3	0.2	5.4	2.5	2.6	7.4	0.9	3.0	8.3
Δhpt ΔadhE::adhE		±1.8	±0.0	±0.1	±0.4	±1.4	±0.4	±0.0	±0.5	±1.1	±0.2	±0.0	±2.3	±0.2	±0.5	±0.2	±0.1	±0.3	±1.4
LL1161	4	11.4	0.0	2.2	2.0	19.0	1.9	0.0	5.1	3.9	0.8	0.1	3.6	2.1	3.5	6.7	1.0	2.5	7.3
Δhpt ΔadhE::$adhE^{D494G}$		±0.8	±0.0	±0.1	±0.4	±1.3	±0.3	±0.0	±0.4	±0.9	±0.5	±0.0	±0.4	±0.2	±0.2	±0.2	±0.0	±0.1	±1.2
