# Supplementary material for: Metabolic and evolutionary responses of Clostridium thermocellum to genetic interventions aimed at improving ethanol production
Source: Biotechnol Biofuels. 2020 Mar 10;13:40. doi: 10.1186/s13068-020-01680-5 (PMC7063780; doi:10.1186/s13068-020-01680-5)

Figure S3. Evidence for merodiploid arrangement of *pta* locus. Cells were used as a template for a PCR reaction with primers XD505 (5'- GGAACAATAAGCACAACCA-3') and XD506 (5'- TCAATCCAAAACCTCCCA-3'). The wild type strain should exhibit a 4,919 bp amplicon, the pta deletion strain should exhibit a 4,044 bp amplicon and the merodiploid strain should exhibit a 7,399 bp amplicon. Based on the gel image, strain LL1004 is wt at the *pta* locus, strain LL1011 has a *pta* deletion, and strains LL1043 and LL1044 are merodiploid.

C. thermocellum pta locus

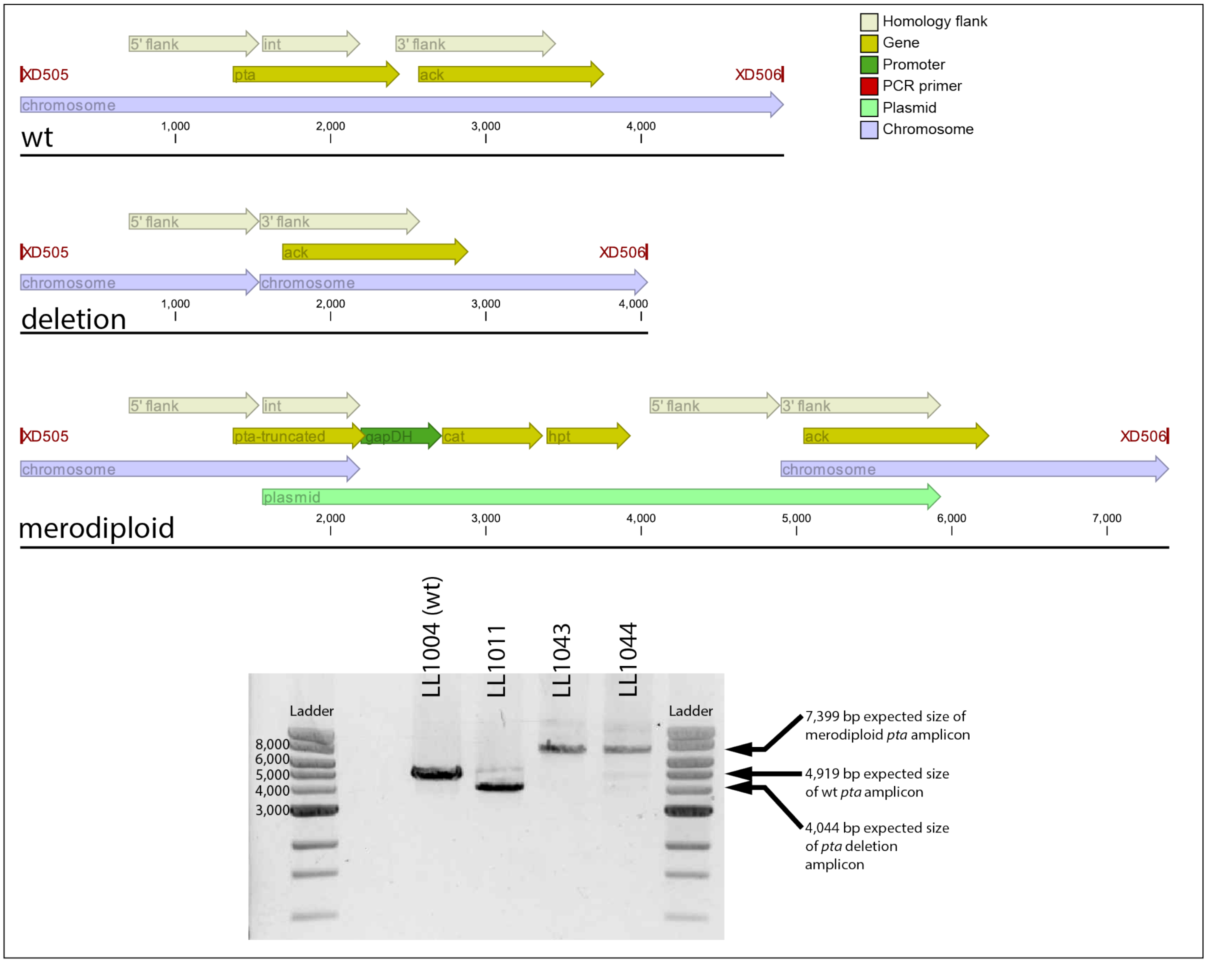

Supplement: Supplementary file 3 — Additional file 3: Figure S3. Evidence for merodiploid arrangement of pta locus. [file 13068_2020_1680_MOESM3_ESM.pdf]
