# Supplementary material for: Metabolic and evolutionary responses of Clostridium thermocellum to genetic interventions aimed at improving ethanol production
Source: Biotechnol Biofuels. 2020 Mar 10;13:40. doi: 10.1186/s13068-020-01680-5 (PMC7063780; doi:10.1186/s13068-020-01680-5)

**Figure S4**. Evidence for wild type arrangement of *gapDH* locus. To rule out the possibility that the *pta* deletion plasmid was integrated at the *gapDH* locus, we amplified the *gapDH* region using primers XD874 (5'-CCTTTGAACTGACCATGTACT-3') and XD875 (5'-TTCGGCCCCTTTCATTCT-3'). In all cases, we found an 836 bp amplicon indicating that the *gapDH* locus is wild type in all strains.

*C. thermocellum gapDH* locus

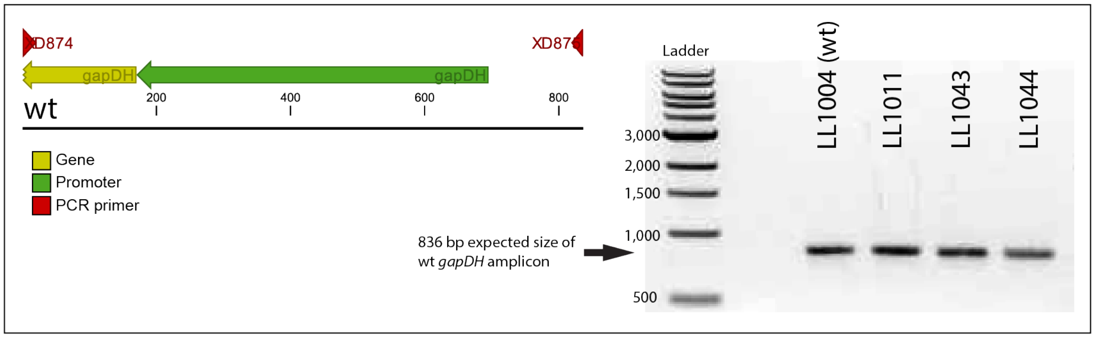

Supplement: Supplementary file 4 — Additional file 4: Figure S4. Evidence for wild type arrangement of gapDH locus. [file 13068_2020_1680_MOESM4_ESM.pdf]
