# Supplementary figures and images for: Metabolic and evolutionary responses of Clostridium thermocellum to genetic interventions aimed at improving ethanol production
Source: Biotechnol Biofuels. 2020 Mar 10;13:40. doi: 10.1186/s13068-020-01680-5 (PMC7063780; doi:10.1186/s13068-020-01680-5)

**Figure S5.** Total number of mutations in each strain, identified by either DNA or RNA sequencing.

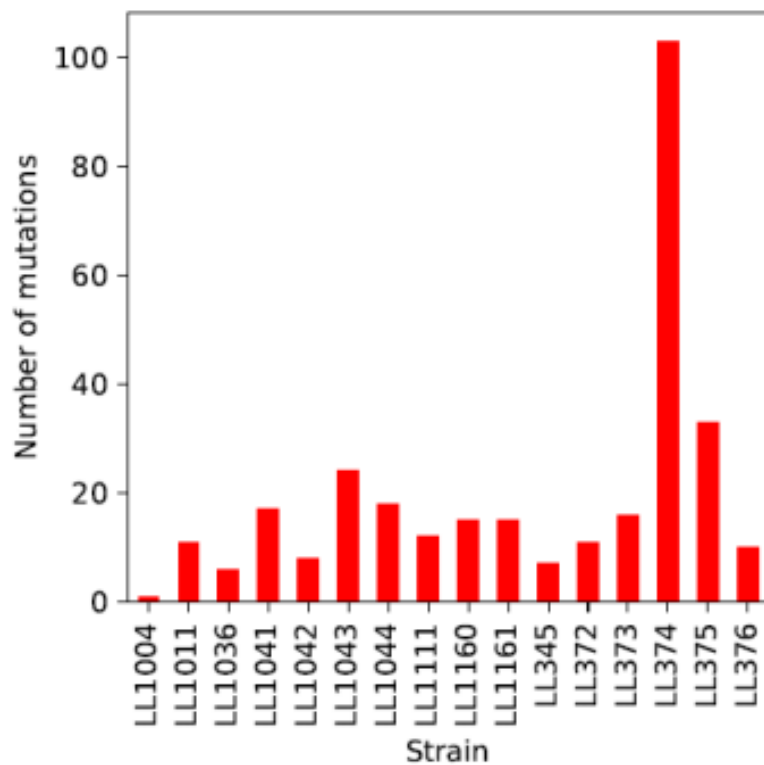

Supplement: Supplementary file 5 — Additional file 5: Figure S5. Total number of mutations in each strain, identified by either DNA or RNA sequencing. [file 13068_2020_1680_MOESM5_ESM.pdf]
