# Supplementary material for: Metabolic and evolutionary responses of Clostridium thermocellum to genetic interventions aimed at improving ethanol production
Source: Biotechnol Biofuels. 2020 Mar 10;13:40. doi: 10.1186/s13068-020-01680-5 (PMC7063780; doi:10.1186/s13068-020-01680-5)
Supplement: Supplementary file 6 — Additional file 6: Supplemental text S6. Discussion of convergent mutations not related to ethanol production. [file 13068_2020_1680_MOESM6_ESM.pdf]

Supplemental text S6: Discussion of convergent mutations not related to ethanol production

To observe mutations that could indicate convergent evolution, we first differentiated mutations that had newly appeared in a strain ('origin mutations') from mutations that had been inherited from the parent strain. (A complete list of mutations is provided in supplementary table S3.) We then identified genes that had accumulated origin mutations in more than one strain, resulting in 10 genes (Figure 6). Mutations corresponding to targeted modifications (*ldh*, *pta, spo0A* and *adhE*^{D494G} in strain LL1161) were removed, since they do not represent convergent evolution. Strains are clustered according to fermentation phenotype (Figure 5). The clustering of origin mutations with fermentation phenotype may give insight into their function (with appropriate caution against overinterpretation of the data, due to the small number of mutations).

In fermentation phenotype 1 (wild type), there are only two genes that have accumulated more than one origin mutation: Clo1313_0908 and Clo1313_1908. Gene Clo1313_0908 is annotated as an adenine phosphoribosyltransferase (*apt*). Mutations in this gene were observed across three of the four fermentation phenotypes (strains LL345, LL376, LL1011 and LL1111), suggesting that this mutation is not associated with a particular change in metabolic flux. Furthermore, mutations in this gene have been observed in other strains of *C. thermocellum* with mutation that have nothing to do with lactate, acetate and ethanol production (for example, LacI regulator deletions [1]). Phosphoribosyltransferase enzymes add a ribose-5-phosphate group to a purine base to create a nucleoside. In *C. thermocellum*, the hypoxanthine phosphoribosyltransferase

(*hpt*) gene is used as a counter-selection marker with 8-azahypoxanthine (8AZH) [2]. It is possible that the *apt* gene has side-activity against 8AZH, and that mutations in this gene reduce 8AZH toxicity.

Gene Clo1313_1908 is annotated as a ErfK/YbiS/YcfS/YnhG family protein (recently re-classified as YkuD) that is thought to act as an L,D-transpeptidase. This mutation is the only one to show up in the same location (i.e. G71V) in three different strains. The only other mutation that shows up in an identical location in more than one strain is the *adhE* D494G mutation, and that only appeared in two different strains. Very little is known about the YkuD protein, although in some organisms it is associated with beta-lactam antibiotic resistance [3].

In fermentation phenotype 2 (ethanol, pyruvate and amino acids), there are two genes with more than one origin mutation: Clo1313_0909 and Clo1313_2832. Clo1313_0909 is annotated as a RelA/SpoT domain protein. In *Escherichia coli*, the *relA* and *spoT* genes are responsible for the “stringent response” whereby the level of guanosine 5,3 bipyrophosphate (ppGpp) is increased, downregulating the translation apparatus and upregulating biosynthesis of amino acids. Since high levels of ppGpp are associated with a variety of physiological stresses, of which the deletion of *pta* may be one, mutations in Clo1313_0909 likely serve the purpose of attenuating the stringent response in *C. thermocellum*. In *C. thermocellum*, adaptation to high levels of ethanol (i.e. ethanol stress) resulted in an A561G mutation in the *spoT* gene (Cthe_1344) [4]. A deletion of *relA* in *E. coli* has been shown to increase biomass yield [5]. The same effect is not observed in *C. thermocellum*, in strain LL1044, biomass is decreased compared to its parent strain, LL372 (supplemental table S1A). it is possible that the effects of this mutation are masked by the *pta* deletion , which decreases biomass production. Furthermore, strains LL374 and LL375, which are both descended from LL1044, have

additional mutations in Clo1313_0909. In strain LL1044, the Clo1313_0909 mutation is a frame shift at position 575 (out of 725), which would be expected to inactivate the protein. Thus, it is surprising that both of the descendants of LL1044 (LL374 and LL375) have an additional mutation in this gene.

Three of the five strains exhibiting fermentation phenotype 2 have a mutation in gene Clo1313_2832. This gene is annotated as a rho termination factor. The rho termination factor is an essential gene in *E. coli* [6] and is thought to regulate between 20 and 30% of transcription termination events in bacteria [7]. It has three PFAM domains, an N-terminal domain, an RNA-binding domain and an ATPase domain [8]. Mutations S314L, R332C and A342V are all in the RNA-binding domain, the P368S is not part of an annotated domain, and the V462A is in the ATPase domain. The R332C mutation, found in strain LL375, is equivalent to the R102C mutation in *E. coli*, based on a protein sequence alignment. This mutation was observed to appear in 1 out of 11 strains of *E. coli* adapted for fast growth on lactate M9 medium [9]. Re-introduction of the mutation in *E. coli* resulted in a 9% increase in growth rate. Mutations in *rho* have been shown to increase ethanol tolerance in *E. coli* by correcting for an aberrant increase in transcription termination [10]. Strains of *C. thermocellum* specifically adapted for increased ethanol tolerance, however, did not show mutations in Clo1313_2832 (equivalent to Cthe_2174 in *C. thermocellum* strain ATCC27405) [4,11]

1. Wilson CM, Klingeman DM, Schlachter C, Syed MH, Wu C, Guss AM, et al. LacI Transcriptional Regulatory Networks in *Clostridium thermocellum* DSM1313. Appl Environ Microbiol. 2017;83:e02751-16

2. Argyros DA, Tripathi S a, Barrett TF, Rogers SR, Feinberg LF, Olson DG, et al. High ethanol titers from cellulose by using metabolically engineered thermophilic, anaerobic microbes. Appl Environ Microbiol. 2011;77:8288–94. doi:10.1128/AEM.00646-11.
3. Biarrotte-Sorin S, Hugonnet JE, Delfosse V, Mainardi JL, Gutmann L, Arthur M, et al. Crystal Structure of a Novel β-Lactam-insensitive Peptidoglycan Transpeptidase. J Mol Biol. 2006;359:533–8.
4. Shao XJ, Raman B, Zhu MJ, Mielenz JR, Brown SD, Guss AM, et al. Mutant selection and phenotypic and genetic characterization of ethanol-tolerant strains of *Clostridium thermocellum*. Appl Microbiol Biotechnol. 2011;92:641–52.
5. Traxler MF, Summers SM, Nguyen HT, Zacharia VM, Hightower GA, Smith JT, et al. The global, ppGpp-mediated stringent response to amino acid starvation in *Escherichia coli*. Mol Microbiol. 2008;68:1128–48.
6. Das A, Court D, Adhya S. Isolation and characterization of conditional lethal mutants of *Escherichia coli* defective in transcription termination factor rho. Proc Natl Acad Sci U S A. 1976;73:1959–63.
7. Kriner MA, Sevostyanova A, Groisman EA. Learning from the Leaders: Gene Regulation by the Transcription Termination Factor Rho. Trends Biochem Sci. 2016;41:690–9.
8. Finn RD, Coggill P, Eberhardt RY, Eddy SR, Mistry J, Mitchell AL, et al. The Pfam protein families database: towards a more sustainable future. Nucleic Acids Res. 2015;44 December 2015:gkv1344.
9. Conrad TM, Joyce AR, Applebee MK, Barrett CL, Xie B, Gao Y, et al. Whole-genome resequencing of *Escherichia coli* K-12 MG1655 undergoing short-term

laboratory evolution in lactate minimal media reveals flexible selection of adaptive mutations. Genome Biol. 2009;10:1–12.

10. Haft RJF, Keating DH, Schwaegler T, Schwalbach MS, Vinokur J, Tremaine M, et al. Correcting direct effects of ethanol on translation and transcription machinery confers ethanol tolerance in bacteria. Proc Natl Acad Sci. 2014;111:E2576–85.

11. Brown SD, Guss AM, Karpinets T V, Parks JM, Smolin N, Yang S, et al. Mutant alcohol dehydrogenase leads to improved ethanol tolerance in *Clostridium thermocellum*. Proc Natl Acad Sci U S A. 2011;108:13752–7. doi:10.1073/pnas.1102444108.
