# Supplementary material for: Metabolic and evolutionary responses of Clostridium thermocellum to genetic interventions aimed at improving ethanol production
Source: Biotechnol Biofuels. 2020 Mar 10;13:40. doi: 10.1186/s13068-020-01680-5 (PMC7063780; doi:10.1186/s13068-020-01680-5)
Supplement: Supplementary file 10 — Additional file 10: Figure S10. Differential gene expression. [file 13068_2020_1680_MOESM10_ESM.pdf]

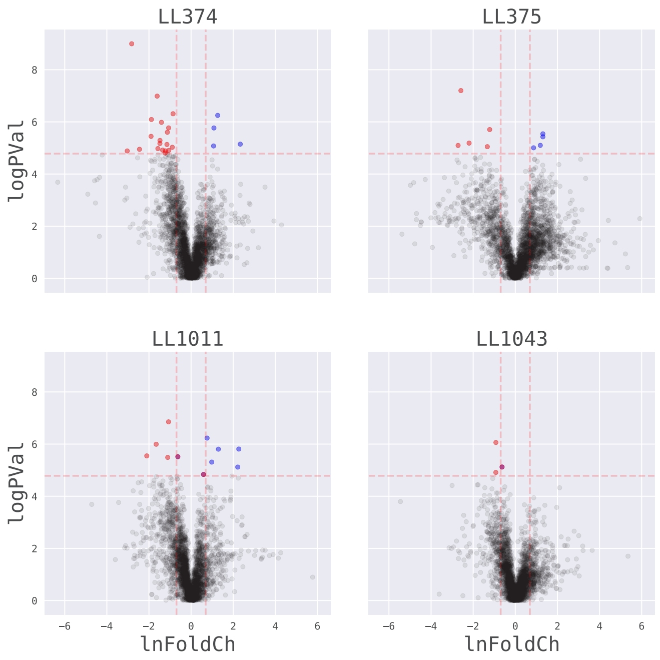

Figure S10. Differential gene expression. Gene expression data for the four evolved *ldh pta* deletion strains (LL347, LL375, LL1011 and LL1043). For each strain, RPKM normalized RNAseq data was compared to a strain that is wild type at the *ldh* and *pta* loci (LL345). Fold change is the ratio of median gene expression.
P-value was calculated based on a 2-sided t-test. The ln(fold-change) was plotted against -log10(p-value). The dashed red lines indicate significance. For fold-change, the cutoff was set to a fold-change of 2 (ln(fold-change) = 0.63). For p-value, a nominal p-value of 0.05 was corrected for 3031 independent tests by the Bonferroni method, resulting in a cutoff of -log10(p-value) = 4.78. In each plot, the upper right and upper left quadrants indicate genes that are significantly over-expressed or under-expressed. Statistics were calculated from 4 RNAseq libraries for each strain. Blue indicates genes that are overexpressed relative to the parent strain (LL345), red indicates genes that are underexpressed.

Strain	geneID	Locus	Common Name	Gene Product	Median expression (log RPKM)	Log fold-change (vs. strain LL345)	Log P-value
LL374	650469753	Clo1313_0913		hypothetical protein	8.72	2.34	5.15
	650470946	Clo1313_2111		RNA polymerase, sigma subunit, RpoX/SigF	3.47	1.26	6.25
	650471138	Clo1313_2319		methylated-DNA/protein-cysteine methyltransferase	12.27	1.08	5.77
	650471140	Clo1313_2322		binding-protein-dependent transport systems inner membrane component	11.59	1.07	5.08
	650469493	Clo1313_0647		hypothetical protein	8.81	-0.86	6.31
	650471132	Clo1313_2310		4Fe-4S ferredoxin iron-sulfur binding domain protein	7.05	-0.89	5.03
	650471229	Clo1313_2417		two component transcriptional regulator, winged helix family	4.59	-1.06	4.91
	650469362	Clo1313_0519		ABC transporter related protein	5.77	-1.07	5.77
	650469939	Clo1313_1101		arsenical-resistance protein	3.65	-1.13	5.60
	650471177	Clo1313_2359		glycerate kinase	4.95	-1.15	5.13
	650469240	Clo1313_0396		transcriptional regulator, LacI family	6.21	-1.22	4.89
	650470842	Clo1313_2006		ADP-ribosylation/Crystallin J1	5.76	-1.23	4.81
	650469995	Clo1313_1157		transglutaminase domain-containing protein	4.25	-1.35	4.92
	650468844	Clo1313_0001		chromosomal replication initiator protein DnaA	7.40	-1.41	5.98
	650471396	Clo1313_2593		MgtC/SapB transporter	5.45	-1.48	5.29
	650469929	Clo1313_1091		hypothetical protein	1.01	-1.48	5.17
	650471066	Clo1313_2238		hypothetical protein	7.33	-1.58	4.98
	650468845	Clo1313_0002		DNA polymerase III, beta subunit (EC 2.7.7.7)	6.81	-1.61	6.99
	650469338	Clo1313_0491		transcriptional regulator, CarD family	7.91	-1.88	6.09
	650470708	Clo1313_1868		transposase mutator type	0.03	-1.90	5.45
	650641722	Clo1313_2362		ID=650641722,locus_tag=Clo1313_2362	2.28	-2.45	4.95
	650468935	Clo1313_0090		S-layer domain-containing protein	6.15	-2.82	9.00
	650469472	Clo1313_0631		transglutaminase domain-containing protein	7.66	-3.03	4.89
LL375	650470117	Clo1313_1275		SSU ribosomal protein S12P methylthiotransferase (EC 2.-.-.-)	9.03	1.31	5.43
	650471478	Clo1313_2687		hypothetical protein	5.12	1.31	5.54
	650470927	Clo1313_2092	pgam	phosphoglycerate mutase (EC 5.4.2.1)	10.39	1.19	5.10
	650470133	Clo1313_1291		signal recognition particle-docking protein FtsY	7.74	0.87	5.01
	650469567	Clo1313_0718		glucose-1-phosphate adenylyltransferase, GlgD subunit	6.75	-1.22	5.71
	650470546	Clo1313_1703		ABC-type bacteriocin transporter	2.48	-1.33	5.05
	650471062	Clo1313_2233		glycoside hydrolase family 5	4.56	-2.19	5.19
	650468935	Clo1313_0090		S-layer domain-containing protein	6.39	-2.58	7.20
	650470657	Clo1313_1813		periplasmic sensor diguanylate cyclase/phosphodiesterase	5.04	-2.72	5.10
LL1011	650471286	Clo1313_2476		cobalamin (vitamin B12) biosynthesis CbiM protein	10.54	2.26	5.81
	650468899	Clo1313_0054		type 3a cellulose-binding domain protein	9.25	2.21	5.12
	650470537	Clo1313_1694		endoglucanase Cel9W	10.33	1.30	5.81
	650470502	Clo1313_1659		endoglucanase Cel9R	9.59	0.98	5.31
	650471780	Clo1313_2992		hypothetical protein	11.03	0.76	6.23
	650469563	Clo1313_0714		Carbohydrate binding family 25	8.88	0.59	4.84
	650469563	Clo1313_0714		Carbohydrate binding family 25	8.88	0.59	4.84
	650471557	Clo1313_2768		Beta propeller domain	7.24	-0.63	5.52
	650471557	Clo1313_2768		Beta propeller domain	7.24	-0.63	5.52
	650470743	Clo1313_1901		NAD+ synthetase	6.42	-1.07	6.85
	650470721	Clo1313_1881	bifur-c	NAD(P)-dependent iron-only hydrogenase catalytic subunit	9.87	-1.11	5.49
	650470384	Clo1313_1540		diaminopimelate decarboxylase	6.92	-1.66	5.99
	650469498	Clo1313_0652		LL-diaminopimelate aminotransferase apoenzyme (EC 2.6.1.83)	5.08	-2.10	5.55
LL1043	650471557	Clo1313_2768		Beta propeller domain	7.24	-0.63	5.12
	650471557	Clo1313_2768		Beta propeller domain	7.24	-0.63	5.12
	650471089	Clo1313_2261		ABC transporter transmembrane region	4.46	-0.92	6.06
	650469929	Clo1313_1091		hypothetical protein	1.57	-0.92	4.91
