# Supplementary material for: Metabolic and evolutionary responses of Clostridium thermocellum to genetic interventions aimed at improving ethanol production
Source: Biotechnol Biofuels. 2020 Mar 10;13:40. doi: 10.1186/s13068-020-01680-5 (PMC7063780; doi:10.1186/s13068-020-01680-5)
Supplement: Supplementary file 11 — Additional file 11: Table S11. Table S11 (A) Overview of the residual cellulose concentration, the cell biomass concentration, main fermentation products, and amino acids produced by the four end-strains (LL374, LL375, LL1011 and LL1043) cultivated on 120 g/L cellulose (Figure 7). (B) Overview of all amino acids produced during fermentation the four end-strains on 120 g/L cellulose (Figure 7). (C) Carbon recovery for the four end-strains on 120 g/L cellulose (Figure 7). [file 13068_2020_1680_MOESM11_ESM.pdf]

Table S11: (A) Overview of the residual cellulose concentration, the cell biomass concentration, main fermentation products, and amino acids produced by the four end-strains (LL374, LL375, LL1011 and LL1043) cultivated on 120 g/L cellulose (Figure 7), the metabolite values shown are the maximum values detected over the duration of fermentation. Values in this table correspond to numerical values shown in Figure 7, but then not rounded

Strain	Total number of replicates	Cellulose utilized (g glucose/L)	Cells (gN/L)	Acetate (g/L)	Formate (g/L)	Lactate (g/L)	Fumarate (g/L)	Pyruvate (g/L)	Ethanol (g/L)	Isobutanol (g/L)	2,3-Butanediol (g/L)	Xylitol (g/L)	Pyroglutamate (g/L)
LL374	2	109.20	0.50	1.86	0.68	1.44	1.18	1.19	24.50	2.26	1.17	0.58	2.75
Δhpt Δspo0A Δldh Δpta::PgapD-cat-hpt adapted		±0.91	±0.00	±0.16	±0.37	±0.79	±0.49	±0.11	±1.10	±0.28	±0.02	±0.20	±0.08
LL375	2	100.80	0.51	1.85	0.78	1.68	2.75	1.25	17.02	1.79	1.50	0.44	3.72
Δhpt Δspo0A Δldh Δpta::PgapD-cat-hpt adapted		±5.53	±0.02	±0.04	±0.81	±0.57	±0.7	±0.21	±1.29	±0.08	±0.02	±0.14	±0.26
LL1011	2	102.60	0.56	1.23	1.04	1.79	0.61	1.04	23.87	3.23	0.40	0.78	0.81
Δhpt Δldh Δpta adapted		±3.83	±0.00	±0.10	±0.01	±0.00	±0.29	±0.00	±2.45	±0.04	±0.30	±0.00	±0.43
LL1043	2	103.20	0.47	3.10	1.02	1.34	0.92	0.87	29.93	5.10	0.81	0.48	1.24
Δhpt Δldh Δpta::PgapD-cat-hpt adapted		±1.64	±0.03	±1.99	±0.15	±1.08	±0.38	±0.16	±0.21	±0.61	±0.37	±0.35	±0.91

Table S11: (B) Overview of all amino acids produced during fermentation the four end-strains on 120 g/L cellulose (Figure 7) in milligram/Liter. The amino acids values shown are the maximum values detected over the duration of fermentation in milligram/Liter. Values in this table correspond to numerical values shown in Figure 7, but then not rounded.

Strain	Total number of replicates	Alanine	Arginine	Asparagine	Aspartic acid	Glutamic acid	Glutamine	Histidine	Isoleucine	Leucine	Lysine	Methionine	Phenylalanine	Proline	Serine	Threonine	Tryptophan	Tyrosine	Valine
LL374	2	2,592.1	8.7	43.7	10.8	725.8	137.8	1.6	606.6	1,144.8	209.1	85.0	7.4	109.6	13.8	162.1	3.9	21.0	6,529.0
Δhpt Δspo0A Δldh Δpta::PgapD-cat-hpt adapted		±74.7	±0.8	±1.5	±1.2	±16.3	±9.8	±0.1	±0.5	±70	±39.9	±2.5	±0.3	±10.1	±0.4	±9.9	±0.5	±0.8	±39.5
LL375	2	1,156.3	12.8	60.9	31.9	727.2	192.1	1.5	451.5	145.1	113.3	84.9	11.9	132.8	24.3	144.5	4.8	44.6	7,041.2
Δhpt Δspo0A Δldh Δpta::PgapD-cat-hpt adapted		±41.7	±4.9	±5.8	±12	±46.6	±5.6	±0.4	±27.1	±9.6	±3.5	±5.8	±2.3	±5.4	±1.8	±17.2	±0.5	±5.7	±5.2
LL1011	2	744.8	24.0	25.4	8.8	434.0	19.2	0.8	697.5	170.0	143.1	85.1	7.9	64.9	53.4	124.8	3.6	17.5	6,569.0
Δhpt Δldh Δpta adapted		±13.5	±2.6	±1.9	±0.3	±31.5	±10.5	±0.1	±1.9	±6.8	±17	±0.1	±0.9	±4	±0.6	±9	±0.1	±2.6	±16.7
LL1043	2	1,162.8	13.4	124.4	72.9	929.6	88.7	3.1	363.2	146.1	338.8	26.7	9.3	67.3	48.7	132.7	6.1	58.1	4,875.2
Δhpt Δldh Δpta::PgapD-cat-hpt adapted		±72.3	±0.1	±4.7	±68.7	±70.7	±0.1	±0.4	±7.6	±62.3	±28	±3.5	±0.6	±0.9	±0.3	±19	±0	±2.9	±251.3

Table S11: (C) Carbon recovery for the four end-strains on 120 g/L cellulose (Figure 7). Values calculated as described in [13] and calculated based on averages from two replicates for each carbon

Strain	Total carbon from measured fermentation products (gram carbon/L)	Total CO_2 # (gram carbon/L)	Total carbon cells ## (gram carbon/L)	Total carbon from amino acids ### (gram carbon/L)	Cellulose carbon solubilized (gram carbon/L)	Total carbon recovery
LL374<br>Δhpt Δspo0A Δldh Δpta::PgapD-cat-hpt adapted	18.86	7.99	1.91	5.53	43.68	78.50%
LL375<br>Δhpt Δspo0A Δldh Δpta::PgapD-cat-hpt adapted	16.02	5.91	1.95	4.94	40.32	71.46%
LL1011<br>Δhpt Δldh Δpta adapted	17.60	7.92	2.14	4.32	41.04	77.92%
LL1043<br>Δhpt Δldh Δpta::PgapD-cat-hpt adapted	22.91	10.32	1.80	3.85	41.28	94.17%

Total CO_2 is based on CO_2 = Acetate + Ethanol – Formate + Valine + 2 * isobutanol on a per mole basis, according to [13].

Total cell carbon is calculated on basis of ratio of 1 gram cell nitrogen : 3.82 gram cell carbon according to [67].

Total carbon from amino acids is calculated based on each measured amino acid, for the maximum of total amino acids measured during the duration of fermentation.
